# Plasma donation rates and infection risk: A multicentre observational study in Denmark

**DOI:** 10.1111/vox.70300

**Published:** 2026-06-02

**Authors:** Kathrine Agergård Kaspersen, István Barkos, Lotte Hindhede, Susan Mikkelsen, Khoa Manh Dinh, Nanna Brøns, Jacob Træholt, Christina Mikkelsen, Jakob Thaning Bay, Ole Birger Vesterager Pedersen, Sisse Rye Ostrowski, Erik Sørensen, Bitten Aagaard, Mie Topholm Bruun, Henrik Hjalgrim, Klaus Rostgaard, Henrik Ullum, Erzsébet Horváth‐Puhó, Christian Erikstrup

**Affiliations:** ^1^ Department of Clinical Immunology Aarhus University Hospital Aarhus Denmark; ^2^ Danish Big Data Centre for Environment and Health (BERTHA) Aarhus University Aarhus C Denmark; ^3^ Department of Clinical Epidemiology and Center for Population Medicine Aarhus University Aarhus N Denmark; ^4^ Department of Clinical Immunology Copenhagen University Hospital, Rigshospitalet Copenhagen Denmark; ^5^ Novo Nordisk Foundation Center for Basic Metabolic Research, Faculty of Health and Medical Science Copenhagen University Copenhagen Denmark; ^6^ Department of Clinical Immunology Zealand University Hospital Køge Denmark; ^7^ Department of Clinical Medicine, Faculty of Health and Medical Sciences University of Copenhagen Copenhagen N Denmark; ^8^ Department of Clinical Immunology Aalborg University Hospital Aalborg Denmark; ^9^ Department of Clinical Immunology Odense University Hospital Odense Denmark; ^10^ Department of Epidemiology Research Statens Serum Institut Copenhagen S Denmark; ^11^ Danish Cancer Society Research Center, Danish Cancer Society Copenhagen Denmark; ^12^ Department of Hematology Copenhagen University Hospital Copenhagen Denmark; ^13^ Statens Serum Institut Copenhagen S Denmark; ^14^ Department of Clinical Medicine Aarhus University Aarhus Denmark

**Keywords:** bias, infection risk, observational study, plasma donation, the healthy donor effect

## Abstract

**Background and Objectives:**

The demand for plasma‐derived medicinal products underscores the need to assess health effects of plasma donation. This study examines the association between plasma donation rates and infection risk in a country with voluntary, non‐remunerated donation.

**Materials and Methods:**

From 2016 to 2024, 45,615 first‐time plasma donors were enrolled in a cohort study with time‐varying exposure. Follow‐up began 90 days after the first plasma donation, with donation rates recalculated every 90 days. Donation rates were categorized as 0, 1 (reference), 2–3, 4–5 or 6–8 donations. Infection was defined using Danish health registers. Survival models were used to estimate hazard ratios (HRs) with 95% confidence intervals (CIs).

**Results:**

The median plasma donation rate was 2 (Q1, 1; Q3, 4). During follow‐up, 15,498 donors experienced an infection. A trend of decreasing HRs for infection was observed with higher donation rates compared with a single donation (0 donations: HR: 0.89, 95% CI: 0.86–0.93; 2–3 donations: HR: 0.85, 95% CI: 0.81–0.89; 4–5 donations: HR: 0.65, 95% CI: 0.59–0.72; and 6–8 donations: HR: 0.48, 95% CI: 0.36–0.62). Findings were consistent across multiple regression models.

**Conclusion:**

In this study, higher plasma donation rates were associated with lower infection risk. Importantly, this inverse association is likely due to bias inherent to observational designs, particularly the internal healthy donor effect, which limits interpretation. Thus, no causal relationship between plasma donation rates and infection risk can be inferred. This underscores the challenges of assessing plasma donation safety using non‐randomized data.

**Highlights**

- In this large observational study of approximately 45,000 voluntary, non‐remunerated plasma donors, higher plasma donation rates were associated with lower observed infection risk.
- Importantly, this inverse association is likely due to bias inherent to observational studies, particularly the internal healthy donor effect, which limits interpretation and precludes causal inference.
- These findings highlight the challenges of evaluating the health effects of plasma donation using non‐randomized data.

## INTRODUCTION

The demand for plasma has increased significantly in recent years, driven by the expanding use of plasma‐derived medicinal products. To meet this growing need, plasma donation activities are being scaled up across Europe [1]. Recently, European guidelines reduced the maximum recommended plasma donation frequency from 33 to 26 times per year [2]. However, this recommendation is based primarily on expert consensus rather than robust evidence. In contrast, paid donors in the United States are permitted to donate up to 104 times annually. While plasma donation is generally considered safe, the long‐term health effects remain under‐researched, with a lack of high‐quality evidence [3].

Immunoglobulin G (IgG) is the most common type of antibody found in blood circulation, and it plays a major role in infection prevention [4]. Several studies have found that donating plasma lowers IgG levels [5, 6, 7], and it is suggested that a plasma donation of 800 mL decreases IgG levels by 13% [8]. Furthermore, the only two randomized controlled trials (RCTs) to date show that donating plasma twice weekly or three times every 2 weeks significantly reduces IgG levels [9, 10]. A recent systematic review, incorporating one of the aforementioned RCTs, an experimental study and six observational studies, supports these findings. However, the clinical impacts of donation‐related IgG depletion on infection risk are unclear. The review also highlighted the ‘healthy donor effect’, a selection bias where donors tend to be healthier than the general population since they must meet strict eligibility criteria [11]. This bias occurs both when individuals enter the donor population and with each subsequent donation, as only those who maintain good health continue donating, referring to the ‘internal healthy donor effect’. Consequently, this effect might mask potential health risks associated with frequent plasma donation [12, 13, 14].

Given the limited literature, establishing a consistent benchmark for plasma donation frequency is challenging due to variations in study designs, small sample sizes and bias inherent with observational studies including the internal healthy donor effect.

Recognizing these challenges, we investigated the association between plasma donation rates and infection risk in a large cohort of plasma donors from a country with non‐remunerated plasma donation. Using nationwide registers with complete follow‐up, this setting allowed comprehensive assessment of infection outcomes during a period of rapid upscaling of plasma collection. To mitigate bias, we applied three different study designs. We hypothesized that higher plasma donation rates would be associated with an increased risk of infection.

## MATERIALS AND METHODS

This study was conducted by combining detailed information on donation history with Danish national health and administrative registers. Denmark has a tax‐supported healthcare system, and every citizen is assigned a unique Danish personal identification number (CPR number) at birth or upon immigration. Using this CPR number, we linked our cohort to the health registers [15].

### Study Population

Donation information was obtained from the Scandinavian Donations and Transfusion (SCANDAT) database in Denmark, which includes blood donation data since 1981 [16].

The study population comprised all first‐time plasma donors registered between 1 January 2016 and 30 April 2024 who were 17–67 years of age at the time of donation. In 2015, the permitted plasma donation frequency in Denmark increased substantially from 4 to 23 donations per year. Consequently, plasma donations prior to 2016 were excluded [17]. Trends in the annual number of plasma donors and donations in Denmark from 2010 to 2024 are shown in Figure S1, illustrating the increase in donation activity over the years.

The age restriction to 67 years aimed to account for potential variations in donation patterns among older donors. Only donors with at least one valid plasma donation were included.

For a subgroup of the donors, more granular data were available for participants included in the Danish Blood Donor Study (DBDS). DBDS is an open cohort and biobank established in 2010. It currently comprises 180,000 Danish blood donors aged 18–74 years [18]. Subgroup analyses with information on self‐reported body mass index (BMI), smoking and health‐related quality of life using the Short Form 12 (SF‐12) questionnaire were possible in the DBDS population.

### Study Designs

**FIGURE 1:**
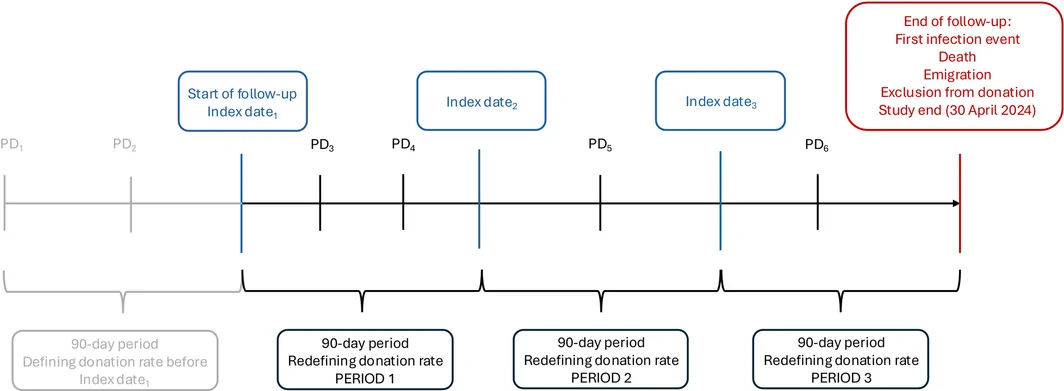
Study design. Plasma donation (PD) rates were analysed in 90‐day intervals using person–time data format. The first 90 days after the initial donation served only to provide an exposure category for follow‐up in the next period. The donation rate was then recalculated for each consecutive 90‐day interval. Use of the donation rate from the previous interval allowed donors to shift between exposure groups over time. For example, the follow‐up PERIOD 2 (between index date_2_ and index date_3_) used the donation rate from the preceding interval, PERIOD 1 (between index date_1_ and index date_2_).

The main study design was a cohort study with time‐varying exposure and time‐dependent confounding variables exploring the association between plasma donation rates and risk of infection. The rate of plasma donation was measured in 90‐day intervals. Follow‐up commenced 90 days after the first plasma donation to have a well‐defined exposure in the first follow‐up period as well as to avoid left truncation and immortal time bias. Plasma donation rates were then recalculated within each subsequent 90‐day interval. Person–time data format was used, with each 90‐day period contributing a separate row to the analysed dataset. The rationale for choosing this approach was to explore the effect of recent and changing plasma donation rates. Furthermore, the approach allowed donors to shift between exposure groups over time and to account for time‐varying covariates. See Figure 1 for study design overview.

To assess the robustness of our findings, we conducted a nested case–control study using incidence density sampling and a cohort study with plasma donation rate as a time‐dependent cumulative exposure over a 1‐year follow‐up. Unlike the main study, this cohort design did not use 90‐day intervals but instead cumulatively counted donation rate over time (details in Supporting Information).

### Exposure

The time‐dependent plasma donation rate was categorized based on the number of donations within each 90‐day interval: 0, 2–3, 4–5 or 6–8 donations versus a single donation (reference group).

### Outcomes

Severe infections were ascertained by relevant hospital discharge diagnoses recorded in the Danish National Patient Register using the International Classification of Diseases, Tenth 10th Revision (ICD‐10) system. To capture mild infections treated by general practitioners, we additionally used data on redeemed antimicrobial prescriptions from the Danish National Prescription Register using the Anatomical Therapeutic Chemical (ATC) classification system. More information is given in Supporting Information. Applying health outcomes from the Danish health registers as a proxy for infections has previously been described [19].

The primary outcome was all‐cause infections, according to both hospital diagnoses and prescriptions. Secondary outcomes included respiratory tract infections, according to prescriptions of antibiotics used to treat respiratory infections. This aspect was particularly interesting, because individuals with low IgG levels have increased susceptibility to these infections [20]. Secondary outcomes also examined all‐cause infection based on hospital diagnoses only and all‐cause infection based on prescriptions only, which together formed the primary outcome.

Separate analysis with hospital diagnoses for respiratory tract infections as outcome was not possible because of the small number of cases.

To further assess the potential impact of the healthy donor bias, we conducted a negative control outcome analysis, in which no association between exposure and outcome is expected. Using the same study design as described above, we examined the association between plasma donation rates and typical emergency department diagnoses, that is, ankle fracture or dislocation in the upper and lower extremities. Injuries that potentially could be related to plasma donation failures (i.e., blood vessel injuries) were excluded.

### Covariates

The following covariates were considered as potential confounders: sex, age, frequency of whole blood donation, distance from the residence to the plasma donation site and season. In a subgroup analysis of DBDS participants, self‐reported BMI, smoking status and health‐related quality of life were additionally included. A directed acyclic graph (Figure S2) and detailed covariate descriptions are provided in Supporting Information. As only 8.3% of the participants had a comorbidity according to the Charlson comorbidity index [21], this variable was not included in the analyses (Table 1).

**TABLE 1:** Characteristics of the cohort.

	Numbers	Percentages
Total	45,615	100
Sex		
Female	21,293	46.7
Male	24,322	53.3
Age at first plasma donation		
17–35	24,540	53.8
36–50	12,858	28.2
51–60	6371	14.0
61–67	1846	4.05
Region		
Capital	6780	14.9
Central Denmark	13,635	29.9
North Denmark	5577	12.2
South Denmark	12,829	28.1
Zealand	6794	14.9
Charlson comorbidity index		
0	41,642	91.3
≥1	3973	8.70
Calendar year (year of first plasma donation)		
2016	5541	12.1
2017	3599	7.89
2018	3544	7.77
2019	3662	8.03
2020	4230	9.27
2021	5382	11.8
2022	6182	13.6
2023	6824	15.0
2024^a^	6651	14.6
Donations during follow‐up		
Whole blood donations, median (Q1; Q3)	0 (0; 1)	
Plasma donations, median (Q1; Q3)	4 (2; 9)	
Infection outcomes during follow‐up		
All‐cause infection, defined by hospital diagnoses and prescriptions (main outcome)	15,498	33.0
Respiratory tract infections, defined by prescriptions	6450	14.1
All‐cause infections, defined by hospital diagnoses	1880	4.12
All‐cause infections, defined by prescriptions	15,115	33.1

*Note*: Numbers with percentages or medians with quartile ranges (Q1; Q3).

^a^ The study period concluded 90 days before the end of follow‐up (30 April 2024).

### Statistical Analyses

The study cohort was characterized by sex, age at baseline, region of residence, Charlson comorbidity index, the calendar year of the first plasma donation and donations during follow‐up. Additionally, we calculated the overall donation rates within 90‐day intervals and by calendar year (2016–2024).

Time‐dependent Cox proportional hazards models were used to estimate crude and adjusted hazard ratios (HRs) with 95% confidence intervals (CIs), comparing outcome rates across categories of time‐updated plasma donations in the preceding 90 days (0, 2–3, 4–5, or 6–8 donations vs. 1 donation). Study participants were followed from their first index date until the first event, death, emigration, date of relevant prescriptions or hospital diagnoses that potentially can lead to permanent exclusion from being a blood donor (i.e., a cancer diagnosis), a maximum of 3 years of follow‐up or 30 April 2024, whichever occurred first.

Crude HRs for all‐cause infection, according to hospital diagnoses and prescriptions as well as respiratory tract infections, were estimated to assess the magnitude of the association without any adjustments. Adjusted HRs were estimated with two multivariable regression models: Model 1 with adjustment for sex, age and whole blood donation frequency (time‐varying), and Model 2 with further adjustment for season (time‐varying) and the distance between residence and plasma donation site (time‐varying).

In a subgroup analysis with DBDS participants, an additional model was based on Model 1 with further adjustments for BMI, smoking status and health‐related quality of life.

The negative control outcome analysis was performed for all‐cause infections according to hospital diagnoses and prescriptions (main outcome).

All analyses were performed in R version 4.4.0 (2024‐04‐24); see Supporting Information for package details.

### Ethics statement

The DBDS was approved by the Central Denmark (1–10‐72‐95‐13) and Zealand (SJ‐740) Regional Committees on Health Research Ethics and the Data Protection Agency (P‐2019–99). SCANDAT was approved by the Data Protection Agency (2008–54‐0472).

## RESULTS

In total, 54,277 plasma donors were identified in SCANDAT during the study period. After application of the eligibility criteria—including at least one 90‐day period ending before 1 May 2024, at least one 90‐day period after the first 90‐day interval, a donation date beyond the first 90‐day interval, age 17–67 years and no infections during the first 90‐day interval—the final study population comprised 45,615 donors. Subgroup analyses using DBDS participants comprised 12,202 donors.

Cohort characteristics are shown in Table 1. The 45,615 included donors had a median follow‐up time of 1.97 years (Q1, 0.97; Q3, 2.95). As expected, males tended to donate more frequently than females. Overall, 15,498 donors experienced an infection, as determined by hospital infection diagnoses and antimicrobial prescriptions.

The overall plasma donation rates for 90‐day intervals and calendar years are shown in Tables S1 and S2. The donation rate declined after the first 90‐day period, and the median decreased from two donations in the first 90‐day period before start of follow‐up to zero donations in the subsequent 11 periods. Donors in the 99th percentile (corresponding to the top 1%) persistently made five donations per period across all intervals (Table S1). Across calendar years (2016–2024), the median annual number of donations was 2. In contrast, among donors in the 99th percentile, the annual number of donations increased from 9 in 2016 to 19 in 2023. The year 2024 was excluded because it did not constitute a full calendar year according to the study design (Table S2).

Characteristics of the subgroup of donors included in DBDS are shown in Table S3.

**FIGURE 2:**
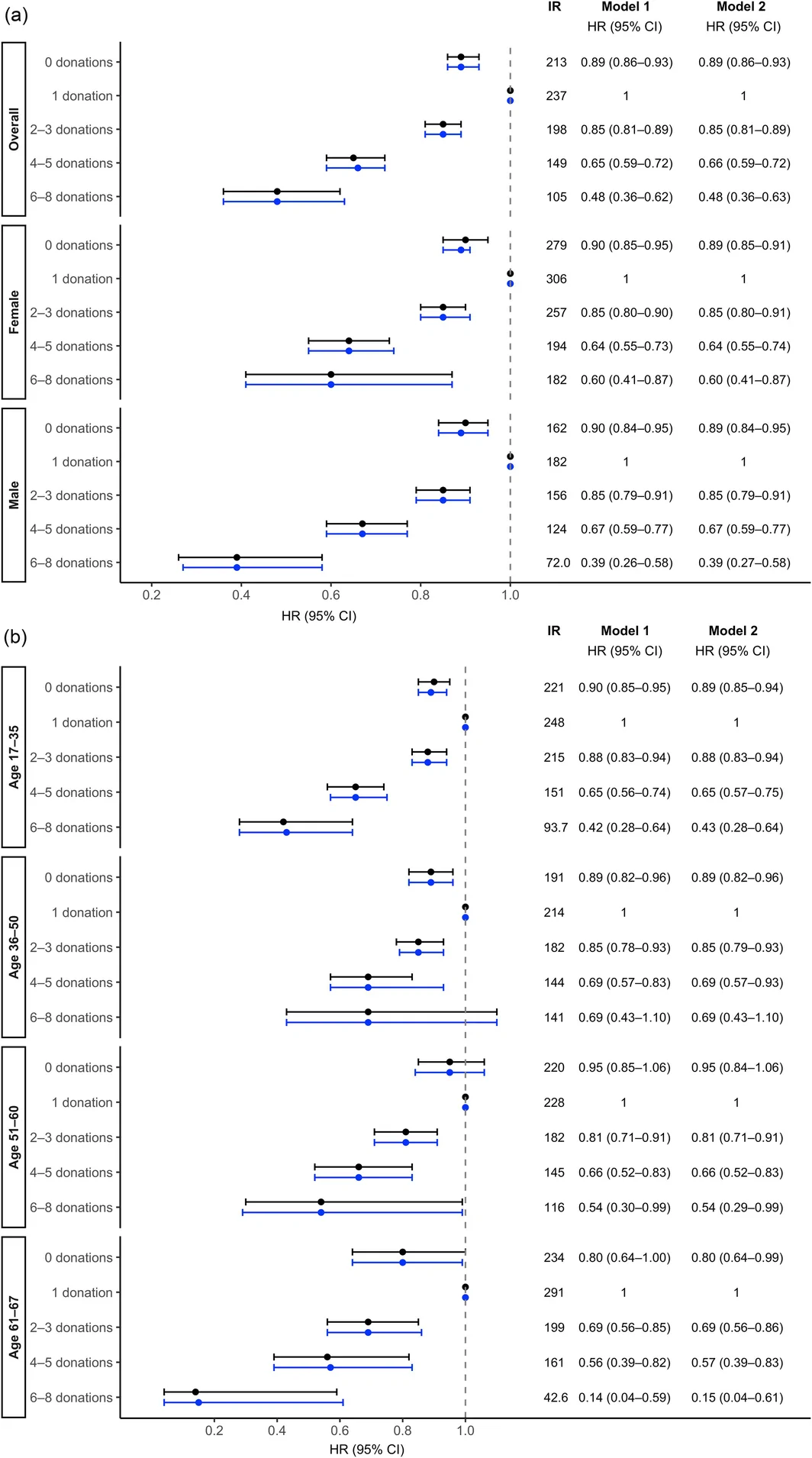
(a) Plasma donation rates and all‐cause infections, overall and by sex. Cox proportional hazard analyses were used to calculate hazard ratios (HRs) with 95% confidence intervals (95% CIs). All‐cause infections were based on both hospital diagnoses and prescriptions. Model 1 (black): Adjusted for sex, age and whole blood donation. Model 2 (blue): Model 1 with further adjustments for distance between residence and the plasma donation site and season. (b) Plasma donation rates and all‐cause infections by age groups. Cox proportional hazard analyses were used to calculate HRs with 95% CIs. All‐cause infections were based on both hospital diagnoses and prescriptions. Model 1 (black): Adjusted for sex, age, and whole blood donation. Model 2 (blue): Model 1 with further adjustments for distance between residence and the plasma donation site and season. IR, incidence rate per 1000 person‐years.

Associations between plasma donation rates and all‐cause infections, overall and stratified by sex and age groups, are presented in Figure 2a,b. A consistent trend of decreasing HRs for infection was observed with higher donation rates (Model 1: 2–3 donations, HR: 0.85, 95% CI: 0.81–0.89; 4–5 donations: HR: 0.65, 95% CI: 0.59–0.72; and 6–8 donations: HR: 0.48, 95% CI: 0.36–0.62) than with one donation in the analysed period. Compared with 1 donation, 0 donations and 2–3 donations yielded similar estimates (HR: 0.85, 95% CI: 0.86–0.93). The findings were consistent across sex and age groups. For all‐cause infections, the effect estimates were lowest in the oldest age group in Model 1 (61–67 years) with 6–8 donations (HR: 0.14, 95% CI: 0.04–0.59) and in males with 6–8 donations (HR: 0.39, 95% CI: 0.26–0.58).

**FIGURE 3:**
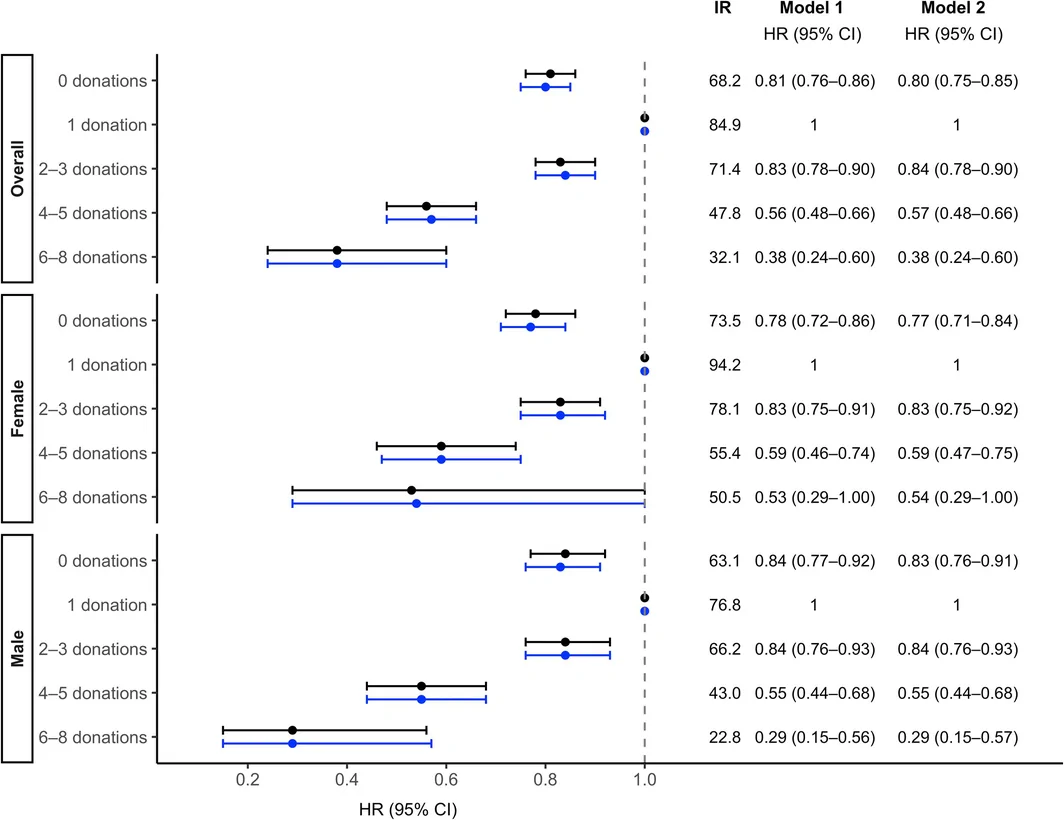
Plasma donation rates and respiratory tract infections, overall and by sex. Cox proportional hazard analyses were used to calculate hazard ratios (HRs) with 95% confidence intervals (95% CIs). Respiratory tract infections were based on prescriptions of antibiotics. Model 1 (black): Adjusted for sex, age and whole blood donation. Model 2 (blue): Model 1 with further adjustments for distance between residence and the plasma donation site and season. IR, incidence rate per 1000 person‐years.

Similar trends were observed for the secondary outcomes (see Figure 3 for respiratory tract infections). The lowest HRs were observed for donors with 6–8 donations (Model 1, both sexes: HR: 0.38, 95% CI: 0.24–0.60; females: HR: 0.53, 95% CI: 0.29–1.00; males: HR: 0.29, 95% CI: 0.15–0.56).

Estimates for other secondary outcomes, including hospital‐treated infections (Figure S3) and antimicrobial prescriptions (Figure S4), showed a similar but weaker effect than observed for respiratory tract infections.

In general, adjustments for the different covariates did not alter the findings (comparison of Model 1 and Model 2 in Figures 2 and 3 and Figures S3 and S4). Moreover, crude HRs yielded comparable estimates (data not shown).

The findings remained robust even in the subgroup analysis with DBDS participants (Figure S5).

In total, 6379 cases were identified for the negative control outcome analysis. Findings showed a similar pattern as for the other analyses, with HRs for emergency department diagnoses decreasing at higher donation rates (Figure S6).

Findings from the nested case–control study and a cohort study with a cumulative exposure measured over a 1‐year follow‐up period are described in Supporting Information. Briefly, the effects were largely consistent with those observed in the main study. However, in the nested case–control study, a slight increase in infection risk was observed for donors who donated only a few times, that is, once within a 2‐year period—a finding difficult to interpret. Both studies revealed a strong trend towards lower infection risk with more donations.

## DISCUSSION

To our knowledge, this study is one of the first to investigate the association between plasma donation rates and short‐term infection risk using high‐quality, register‐based data in a large donor population.

While our data suggest lower infection risk with frequent donation, we do not attribute this to a protective effect from plasma donation. Instead, the internal healthy donor effect likely explains these findings. Despite applying three distinct study designs to mitigate bias, the findings remained consistent.

Although our cohort was large (~45,000 donors), it is modest relative to the millions of global plasma donations annually. Denmark has only moderate plasma donation rates, allowing up to 26 donations per year according to Danish guidelines [17]. In our cohort, the top 1% of donors had a maximum of 19 donations in 2023, corresponding to just under three quarters of the recommended limit. Thus, our findings may not be extrapolatable to settings with higher donation rates.

RCTs provide the highest quality evidence but are rare due to cost. A recent Belgian RCT (63 male donors) showed that donating twice weekly for 12 weeks (per US guidelines) decreased IgG by 38%, while donating three times monthly (per European guidelines) led to an 11% reduction, with the steepest decline in the first 6 weeks [9]. Another recent Norwegian RCT (120 male donors) showed that donating three times every 2 weeks for 16 weeks decreased IgG by 28%, while donating once every 2 weeks led to an 8.9% reduction [10]. However, small sample sizes and exclusion of female donors limit these studies' generalizability and power to assess health outcomes.

A prospective, controlled, multicentre study by Moog et al. [5] assessed adverse events, including infections, in relation to IgG levels below the threshold of 6 g/L. Their study found no evidence of an association between low IgG levels and an increased risk of infections or other adverse events. However, differences in study design and exposure definition limit direct comparability with the present study.

Plasma donation policies vary. Countries with remunerated systems, like the United States and Germany, allow up to 104 and 60 donations per year, respectively. Non‐remunerated systems, such as Denmark, France, Italy and the Netherlands, impose stricter limits, typically allowing plasma donations once every 2 weeks [22].

Although the US Food and Drug Administration permits up to two plasma donations per week, evidence supporting the safety of this frequency is limited and methodologically inconsistent. Observational studies have suggested that repeated plasma donation is safe for donors; however, these studies have important methodological limitations, including selection bias related to the healthy donor effect. Consequently, this evidence is of very low quality and does not robustly support claims of donor safety at such high donation frequencies [23].

Donors in this study were non‐remunerated volunteers meeting stringent eligibility criteria, resulting in a donor cohort generally healthier than the general population. This is reflected in better self‐reported health status [24] and lower all‐cause mortality [12], consistent with the healthy donor effect and additional selection within the donor population referred to as the internal healthy donor effect. Temporary deferral of donors with recent infections and the requirement for good health at the time of donation likely contribute to this selection, such that frequent donors represent a particularly healthy subset. In our study, however, donation frequency was assessed within each 90‐day interval, whereas infections were assessed in the subsequent 90‐day interval. The temporal separation between exposure and outcome suggests that the observed inverse association reflects a selection effect that extends beyond the donation interval itself.

We applied several approaches to mitigate the internal healthy donor effect and other biases inherent to observational studies, including adjustment for BMI, smoking status, health‐related quality of life, whole blood donation frequency, distance between residence and the donation centre and seasonal variations. Despite our efforts, we were unable to fully account for bias, as further supported by the negative control outcome analysis. This illustrates that further methodological development is needed in this field. Alternative designs, such as self‐controlled approaches, may help reduce between‐person confounding but are challenging to implement in this context due to heterogeneous and time‐varying donation patterns, which complicate the definition of appropriate risk and reference windows.

Donors with zero donations in a 90‐day interval likely represent a heterogeneous group, including donors who temporarily paused donation, initiated donation but did not return or simply donated later in a subsequent interval. This makes them unsuitable as a reference category. Unfortunately, we lacked data on the reasons for donation discontinuation, limiting insight into non‐return characteristics.

Similar low estimates were observed when comparing donors with zero donations to those with 2–3 donations. This finding is difficult to explain and, again, suggests that the observed associations may reflect underlying selection processes or residual confounding rather than causal effects of donation frequency. Estimates were even lower among donors with 4–8 donations and those aged 61–67 years, further supporting a pronounced internal healthy donor effect.

Donation frequency was highest in the first 90‐day period, with a median of two donations, and declined thereafter, with a median of zero donations in subsequent intervals. This suggests that many donors do not donate consistently over time, whereas a subset maintains higher donation frequencies, as reflected by the stable upper percentiles (Table S1).

This study has several strengths and limitations. A major strength of this study is the large cohort size and the use of well‐established national registers. In addition, the cohort reflects typical donation rates in a country that is well under way towards self‐sufficiency with non‐remunerated donors. Additionally, questionnaire data from the DBDS enabled adjustment of lifestyle factors and health‐related confounders, thus enhancing the robustness of the study. Despite this strength, we had access to only a limited set of health‐related confounders; that is, why residual confounding might persist. Moreover, a key limitation is the lack of questionnaire data aligned with the start of each follow‐up period.

Despite employing multiple designs, the internal healthy donor effect persisted and remains the most plausible explanation for our findings. Nevertheless, other factors should be considered. Residual confounding and measurement bias, including inaccuracies in self‐reported data or misclassification of infection outcomes, may have influenced the observed associations. In addition, infections may be underestimated, as mild cases (e.g., upper respiratory tract viral infections) that do not require prescription treatment or are managed with over‐the‐counter medications are not captured.

Minor limitations include a major shift in plasma donation protocols in 2012 as well as the use of municipality codes instead of precise home address data to calculate distance to the donation site.

Importantly, our findings are based on Danish plasma donation practices and therefore reflect a real‐life setting, where lower donation rates may allow sufficient IgG recovery, limiting generalizability to countries with higher donation thresholds.

Although plasma donation is generally safe, long‐term prospective studies are needed to refine guidelines and ensure donor safety [25, 26].

In conclusion, we found no consistent evidence for an association between plasma donation rates and infection risk. Our findings suggested an inverse association, with higher plasma donation rates associated with lower infection risk; however, these associations likely reflect bias inherent to observational designs, particularly the internal healthy donor effect, limiting interpretation and precluding causal inference. Findings were consistent across different study designs, underscoring the challenges and limited validity of observational approaches for evaluating the health effects of plasma donation.

## CONFLICT OF INTEREST STATEMENT

The Department of Clinical Epidemiology, Aarhus University, receives funding for other studies from companies in the form of research grants to (and administered by) Aarhus University. None of these studies has any relation to the present study. C.E. has received funding for other studies from Abbott Diagnostics and Novo Nordisk, but no personal fees.

## Supporting information


**Figure S1.** Number of plasma donations by year, Denmark, 2010–2024.
**Figure S2**. Directed acyclic graph.
**Table S1**. Plasma donation rates during the 90‐day intervals.
**Table S2**. Annual plasma donation rates for the study cohort.
**Figure S3**. Plasma donation rates and hospital diagnoses for infections, overall and by sex.
**Figure S4**. Plasma donation rates and prescriptions of antimicrobials, overall and by sex.
**Table S3**. Subgroup analysis: Characteristics of donors included in the DBDS.
**Figure S5**. Subgroup analysis: Plasma donation rates and all‐cause infections with further adjustment for BMI, smoking, and health‐related quality of life.
**Figure S6**. Negative control outcome analysis: Plasma donation rates and emergency department diagnoses, overall.
**Table S4**. Characteristics of cases and controls.
**Table S5**. Plasma donations rates and all‐cause infections.
**Table S6**. Plasma donation rates and respiratory tract infections.
**Table S7**. Plasma donation rates and hospital diagnoses for infections.
**Table S8**. Plasma donation rates and prescriptions of antimicrobials.
**Table S9**. Characteristics of the first‐time plasma donors.
**Table S10**. Plasma donation rates and all‐cause infections, overall and by sex.
**Table S11**. Plasma donation rates and respiratory tract infections, overall and by sex.
**Table S12**. Plasma donation rates and hospital diagnoses for infections, overall and by sex.
**Table S13**. Plasma donation rates and prescriptions of antimicrobials, overall and by sex.

## Data Availability

The DBDS is a platform for studies performed by the Danish blood centres and collaborators. Additional information can be found at http://www.dbds.dk. We invite researchers to collaborate by contacting the steering committee (info@dbds.dk). Data access requires that projects and applicants obtain permission from the Regional Committees on Health Research Ethics and the Danish Data Protection Agency (http://www.datatilsynet.dk). International researchers may gain data access if governed by a Danish research institution because approval and data access are required.
