# Supplementary material for: Plasma donation rates and infection risk: A multicentre observational study in Denmark
Source: Vox Sang. 2026 Jun 2;121(9):1312–21. doi: 10.1111/vox.70300 (PMC13563483; doi:10.1111/vox.70300)
Supplement: Supplementary file 1 [file VOX-121-1312-s001.pdf]

Supplementary Material

Plasma donation rates and infection risk: A multicentre observational study in Denmark

Kaspersen et al.

Table of Contents

The cohort study with time-varying exposures 3

Figure S1. Number of plasma donations by year, Denmark, 2010–2024 3

Figure S2. Directed acyclic graph 4

Covariates 5

Statistical analyses 6

Table S1. Plasma donation rates during the 90-day intervals 6

Table S2. Annual plasma donation rates for the study cohort 6

Figure S3. Plasma donation rates and hospital diagnoses for infections, overall and by sex 7

Figure S4. Plasma donation rates and prescriptions of antimicrobials, overall and by sex 8

Table S3. Subgroup analysis: Characteristics of donors included in the DBDS 9

Figure S5. Subgroup analysis: Plasma donation rates and all-cause infections with further adjustment for BMI, smoking, and health-related quality of life 10

Figure S6. Negative control outcome analysis: Plasma donation rates and emergency department diagnoses, overall 11

The nested case-control study 12

Method 12

Study population 12

Cases and controls 12

Exposure 12

Statistical analyses 13

Results 13

Table S4. Characteristics of cases and controls 14
Conclusion 15
Table S5. Plasma donations rates and all-cause infections 16
Table S6. Plasma donation rates and respiratory tract infections 17
Table S7. Plasma donation rates and hospital diagnoses for infections 18
Table S8. Plasma donation rates and prescriptions of antimicrobials 19
The cohort study with cumulative exposure rate measured over a one-year follow-up period 20
Method 20
Study population 20
Exposure and outcomes 20
Statistical analyses 20
Results 21
Conclusion 21
Table S9: Characteristics of the first-time plasma donors 22
Table S10: Plasma donation rates and all-cause infections, overall and by sex 23
Table S11: Plasma donation rates and respiratory tract infections, overall and by sex 24
Table S12: Plasma donation rates and hospital diagnoses for infections, overall and by sex 25
Table S13: Plasma donation rates and prescriptions of antimicrobials, overall and by sex 26
Delineation of infections 27
Main outcome 27
Secondary outcomes 31
Negative control outcomes 32
Emergency department diagnoses 32
References 33

The cohort study with time-varying exposures

Figure S1. Number of plasma donations by year, Denmark, 2010–2024

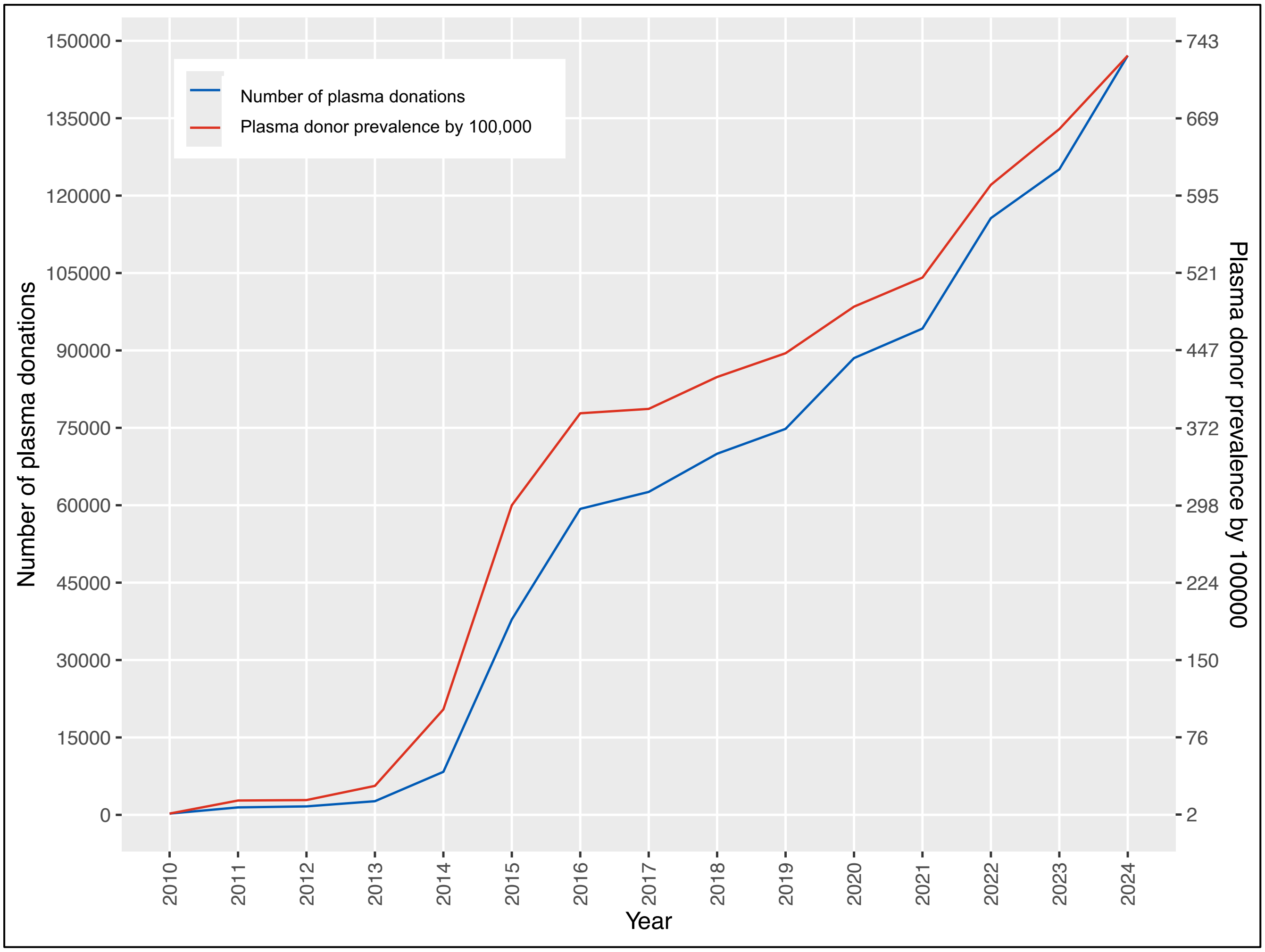

Number of plasma donations by year and plasma donor prevalence by 100,000 inhabitants in Denmark. Plasma donations have increased significantly since 2014–2015, reflecting efforts to achieve self-sufficiency.

Figure S2. Directed acyclic graph

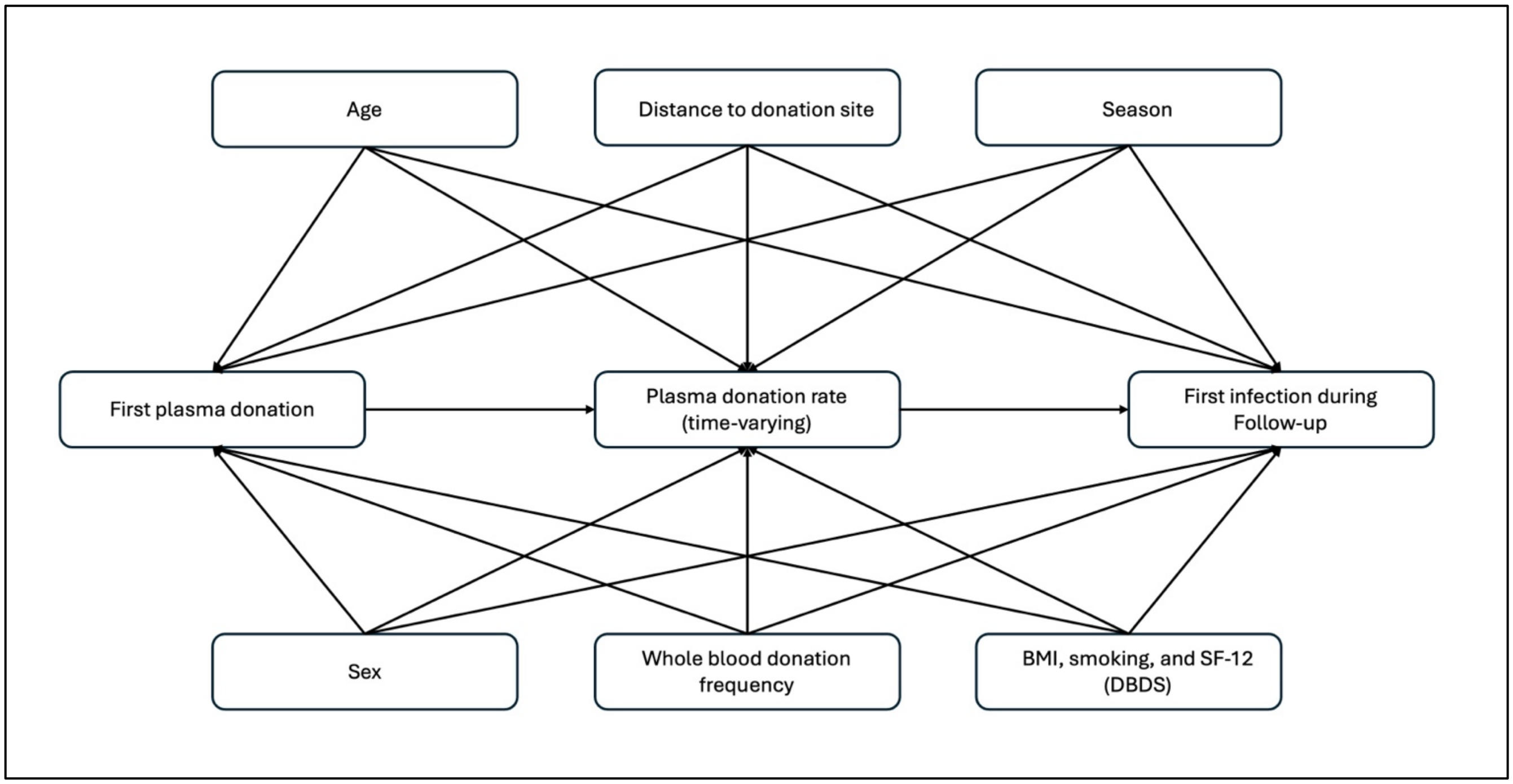

The following covariates were considered as potential confounders: sex, age, frequency of whole blood donations, residential distance from the plasma donation site, and season. For participants in the DBDS, adjustments were additional made for body mass index (BMI), smoking status, and health-related quality of life. The definitions of the covariates are described later in supplementary material.

Covariates

The following covariates were considered potential confounders: sex, age, frequency of whole blood donation, residential distance from the plasma donation site, and season. In a subgroup analysis including DBDS participants, self-reported BMI, smoking status, and health-related quality of life using the SF-12 questionnaire were further considered.

Age at baseline was categorized as: 17–35 (reference group), 36–50, 51–60, and 61–67 years.

Frequency of whole blood donation was defined as a binary indicator variable.

Residential distance from the plasma donation site was defined using municipality codes, categorized as 0–20 km and more than 20 km. This variable was included since residents in rural areas may have different donation habits and medical-seeking behaviours when infected. Furthermore, greater distances might impact donor willingness and capability to go donating.

Season was categorized as April to September and October to March and was included as a potential confounder as it might affect donation frequency but also susceptibility to infections during colder months.

Frequency of whole blood donation, residential distance from the plasma donation site, and season were re-measured during follow-up and treated as time-varying confounders in the regression analyses.

In the subgroup analyses of participants in the DBDS, we additionally adjusted for BMI, smoking status, and health-related quality of life. As described in the main text, DBDS is an open cohort and biobank. Repeat donors, aged 18–74 years, are invited to participate in the study after oral and written information is provided. Donors, who agree to participate, fill out an electronic questionnaire, and their blood samples are stored. Data on BMI, smoking status, and health-related quality of life were extracted from the most recent questionnaires completed within two years of the first index date. The analysis was limited to complete cases, including only donors with non-missing information on these parameters and constituted 12,202 donors out of 45,615 donors included in the main analysis.

We have earlier shown a strong association between obesity and infection[1] in addition to smoking and infection.[2] Similarly, we have shown that active donors report a better health-related quality of life than non-active donors. We speculated that this is also the case *within* the donor pool, which could potentially affect both our exposure and outcome.[3]

BMI and smoking were defined as binary variables (BMI below 30 versus BMI equal to or above 30, and smoker versus non-smoker).

Health-related quality of life was assessed using the SF-12 questionnaire, an abbreviated version of the 36-item short-form health survey (SF-36), which is widely used to describe health-related quality of life in population

surveys. The SF-12 provides a physical (PCS) and mental (MCS) component summary score. Scores range from 0 to 100, with 0 indicating the lowest and 100 the highest level of health. The SF-12 score was included as a continuous variable. Both the SF-36 and SF-12 are valid tools for assessing general health in populations.[4]

Statistical analyses

All analyses were performed in R version 4.4.0 (2024-04-24) using the following libraries: base, broom, data.table, dplyr, epiDisplay, openxlsl, readxl, readr, reshape2, stringr, sqldf, survival, tidyr, tidyverse.

Table S1. Plasma donation rates during the 90-day intervals

90-day period	Q1	Median	Q3	95-percentile	99-percentile	Mean
1 (first 90-day interval)	1	2	2	4	5	1.90
2	0	0	1	3	5	0.87
3	0	0	1	3	5	0.79
4	0	0	1	3	5	0.77
5	0	0	1	3	5	0.73
6	0	0	1	3	5	0.69
7	0	0	1	3	5	0.67
8	0	0	1	3	5	0.66
9	0	0	1	3	5	0.65
10	0	0	1	3	5	0.63
11	0	0	1	3	5	0.62
12	0	0	1	3	5	0.62

Plasma donation rates during the 90-day intervals for the study cohort.

Table S2. Annual plasma donation rates for the study cohort

Year	Q1	Median	Q3	95-percentile	99-percentile	Mean
2016	1	2	4	7	9	2.69
2017	1	2	4	9	12	3.21
2018	1	2	5	9	13	3.42
2019	1	2	4	9	12	3.18
2020	1	2	4	10	16	3.40
2021	1	2	4	10	16	3.31
2022	1	2	5	11	18	3.62
2023	1	2	4	11	19	3.60
2024[a]	1	3	5	12	18	3.94

Annual plasma donation rates for the study cohort. [a]The study period concluded 90 days before end of follow-up (30 April 2024).

Figure S3. Plasma donation rates and hospital diagnoses for infections, overall and by sex

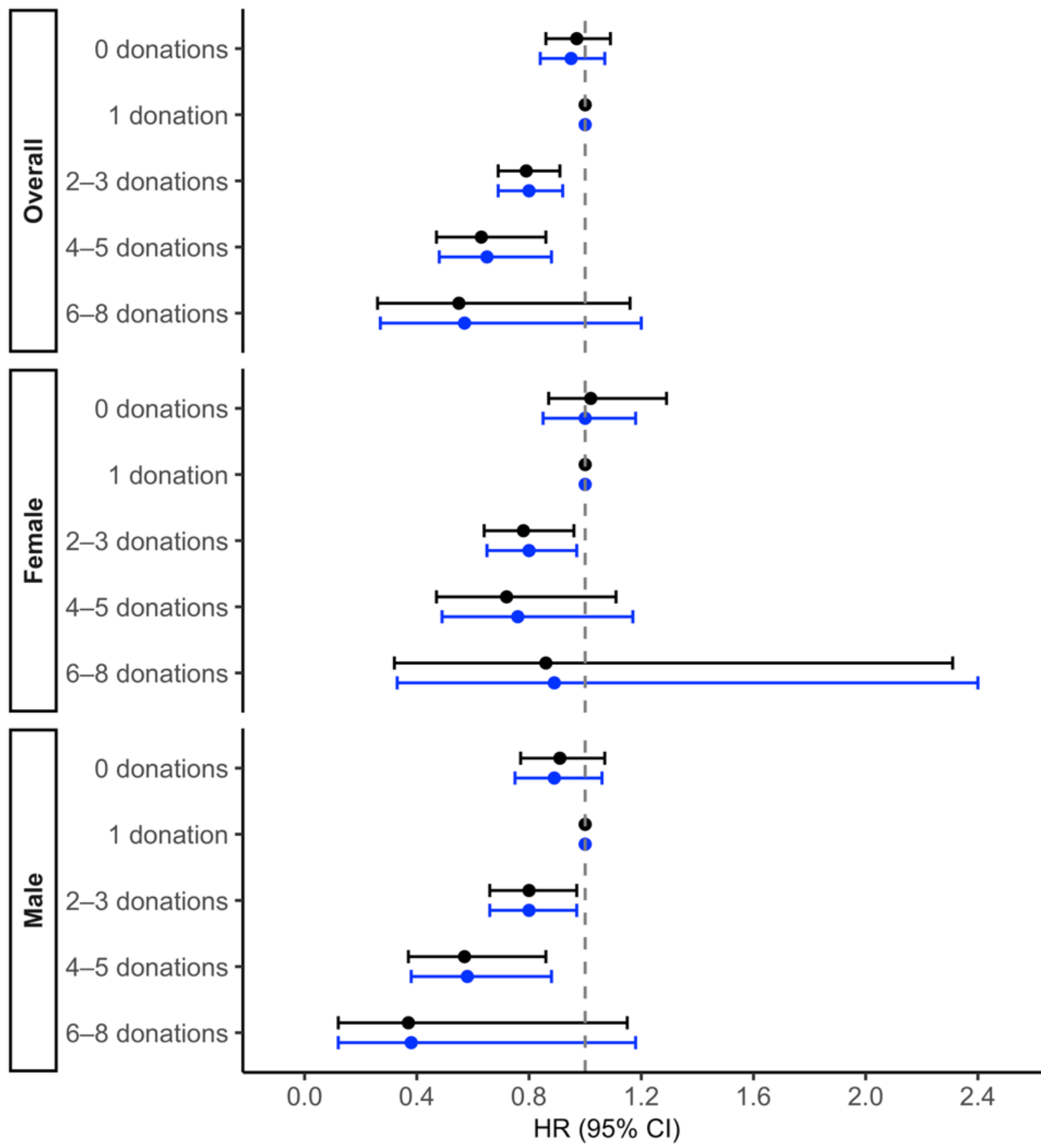

		IR	Model 1 HR (95% CI)	Model 2 HR (95% CI)
Overall	0 donations	20.2	0.97 (0.86–1.09)	0.95 (0.84–1.07)
	1 donation	20.6	1	1
	2–3 donations	16.2	0.79 (0.69–0.91)	0.80 (0.69–0.92)
	4–5 donations	12.9	0.63 (0.47–0.86)	0.65 (0.48–0.88)
	6–8 donations	11.1	0.55 (0.26–1.16)	0.57 (0.27–1.20)
Female	0 donations	22.8	1.02 (0.87–1.29)	1.00 (0.85–1.18)
	1 donation	22.0	1	1
	2–3 donations	17.2	0.78 (0.64–0.96)	0.80 (0.65–0.97)
	4–5 donations	15.9	0.72 (0.47–1.11)	0.76 (0.49–1.17)
	6–8 donations	18.7	0.86 (0.32–2.31)	0.89 (0.33–2.40)
Male	0 donations	17.6	0.91 (0.77–1.07)	0.89 (0.75–1.06)
	1 donation	19.3	1	1
	2–3 donations	15.4	0.80 (0.66–0.97)	0.80 (0.66–0.97)
	4–5 donations	11.1	0.57 (0.37–0.86)	0.58 (0.38–0.88)
	6–8 donations	7.2	0.37 (0.12–1.15)	0.38 (0.12–1.18)

Cox proportional hazard analyses were used to calculate hazard ratios (HRs) with 95% confidence intervals (95% CIs). Model 1 (black): adjusted for sex, age, and whole blood donation. Model 2 (blue): model 1 with further adjustments for residential distance from the plasma donation site and season. IR: Incidence rate per 1000 person-years.

Figure S4. Plasma donation rates and prescriptions of antimicrobials, overall and by sex

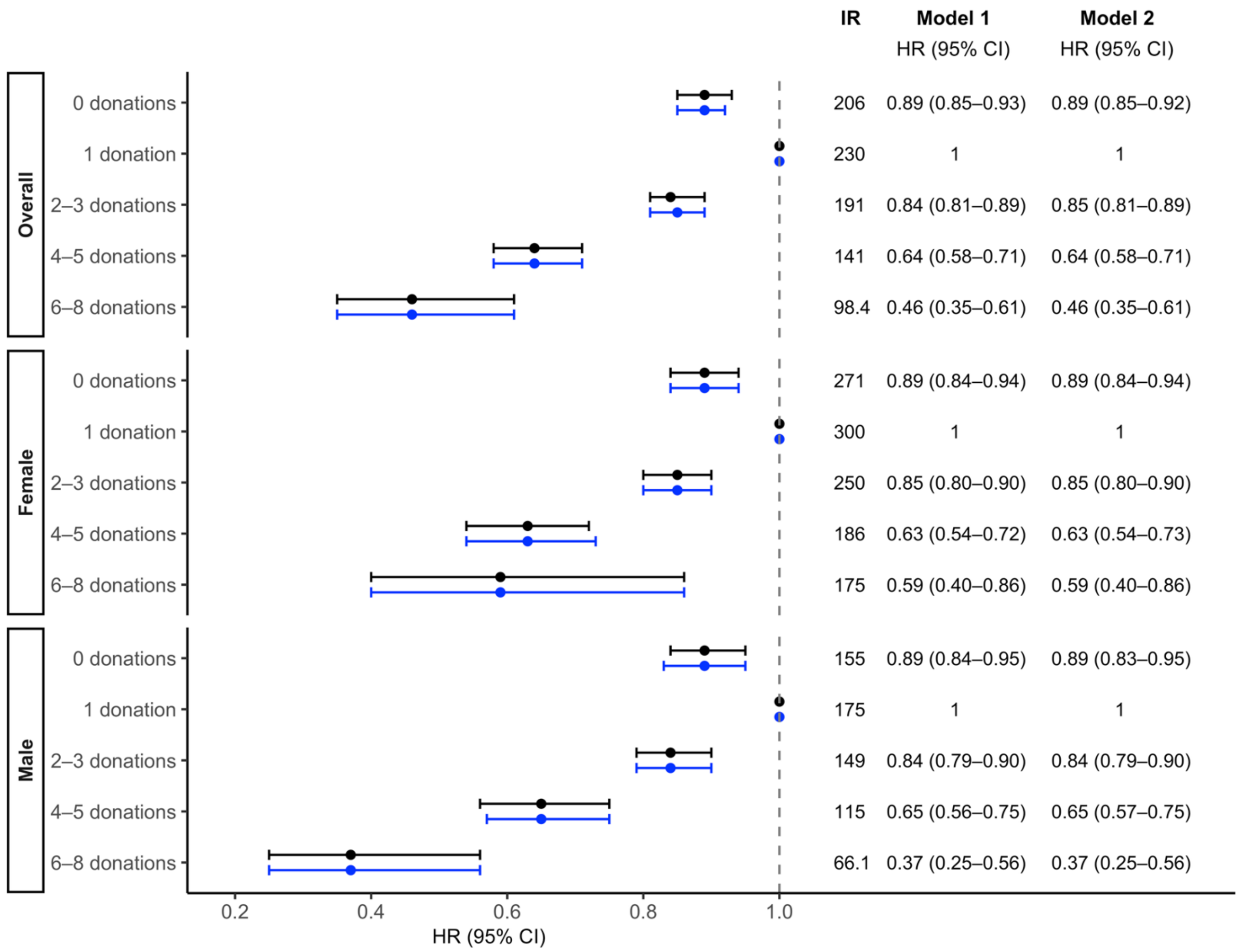

Cox proportional hazard analyses were used to calculate hazard ratios (HRs) with 95% confidence intervals (95% CIs). Model 1 (black): adjusted for sex, age, and whole blood donation. Model 2 (blue): model 1 with further adjustments for residential distance from the plasma donation site and season. IR: Incidence rate per 1000 person-years.

Table S3. Subgroup analysis: Characteristics of donors included in the DBDS

	Numbers	Percentages
**Total**	12,202	100
**Sex**		
Female	5,479	44.9
Male	6,723	55.1
**Age at first-time plasma donation**		
17–35	5,902	48.4
36–50	3,720	30.5
51–60	1,923	15.8
61–67	657	5.38
**Region**		
Capital	2,247	18.4
Central Denmark	3,449	28.3
North Denmark	2,181	17.9
South Denmark	1,596	13.1
Zealand	2,729	22.4
**Calendar year (year of first plasma donation)**		
2016	1,549	12.7
2017	1,632	13.4
2018	1,505	12.3
2019	1,624	13.3
2020	958	7.85
2021	1,378	11.3
2022	2,012	16.5
2023	1,233	10.1
2024[a]	311	2.55
**Donations during follow-up**		
Whole blood donations, median (Q1; Q3)	0 (0; 1)	
Plasma donations, median (Q1; Q3)	3 (1; 7)	
**Smoking status**		
No	10,662	86.6
Yes	1,540	13.4
**Infection outcomes during follow-up**		
All-cause infection, according to hospital diagnoses and prescriptions	4,487	36.8
**BMI, median**	25.7 (23.5; 28.4)	
**MCS, median**	54.8 (49.0; 57.1)	
**PCS, median**	56.6 (54.1; 57.9)	

Numbers with percentages or medians with quartile ranges (Q1; Q3). [a]The study period concluded 90 days before end of follow-up (30 April 2024). BMI: Body Mass Index; MCS: Mental Component Score; PCS: Physical Component Score.

Figure S5. Subgroup analysis: Plasma donation rates and all-cause infections with further adjustment for BMI, smoking, and health-related quality of life

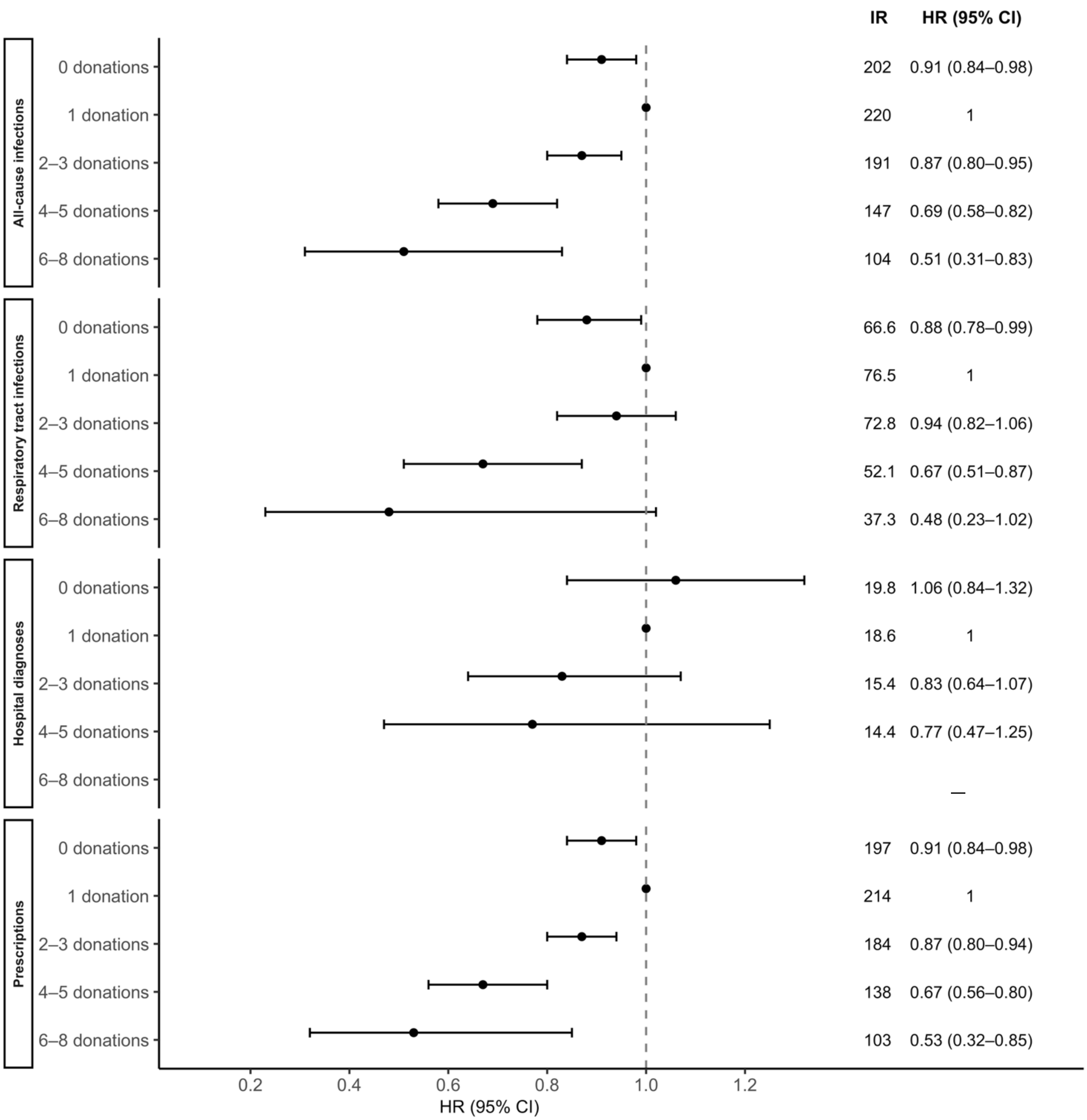

Cox proportional hazard analyses were used to calculate hazard ratios (HRs) with 95% confidence intervals (95% CIs). Adjusted for sex, age, whole blood donation, body mass index, smoking, and health-related quality of life. Analyses limited to participants in the Danish Blood Donor Study (DBDS). HRs for both sexes are shown. IR: Incidence rate per 1000 person-years.

Figure S6. Negative control outcome analysis: Plasma donation rates and emergency department diagnoses, overall

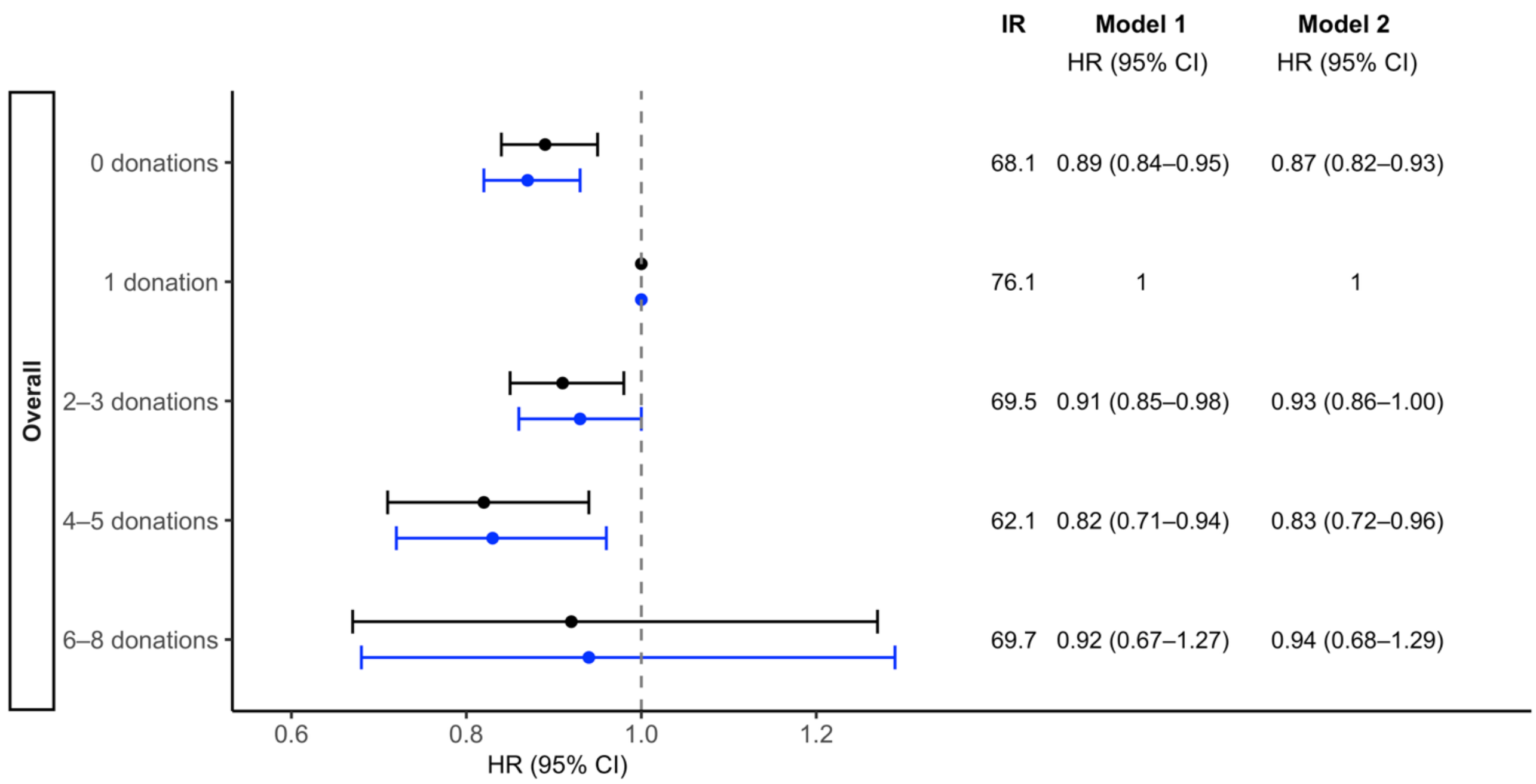

Cox proportional hazard analyses were used to calculate hazard ratios (HRs) with 95% confidence intervals (95% CIs). Model 1 (black): adjusted for sex, age, and whole blood donation. Model 2 (blue): model 1 with further adjustments for residential distance from the plasma donation site and season. IR: Incidence rate per 1000 person-years.

The nested case-control study

In this section, we describe the nested case-control study.

Method

Study population

As described in the main paper.

Eligibility criteria for study entry included: being a first-time plasma donor between 1 January 2016 and 30 April 2024, 17–67 years of age, a minimum of six months' residency in Denmark before the case-control date (index date), no Charlson Comorbidity Index score[5] recorded within five years before the first plasma donation, no infection diagnosis within one year prior to the first plasma donation, no redeemed prescription for any infection within two months before the first plasma donation, and no Charlson Comorbidity Index score recorded after the first plasma donation and before the index date.
R package comorbidity version 0.5.3 was used for calculating the Charlson Comorbidity Index.[6]

Cases and controls

Incident cases were defined as donors with a first-time diagnosis of infection or a first-time redeemed prescription of antimicrobials in the study period. The definition of infections and subgroups are described in the main paper: the primary outcome was all-cause infections based on hospital diagnoses and antimicrobial prescriptions. Secondary outcomes included respiratory tract infections based on relevant prescriptions of antibiotics, all-cause infection based on hospital diagnoses only, and all-cause infection based on prescriptions only.
Incidence density sampling with replacement was performed to select controls alive and free of previous infection. Cases and controls were matched 1:1 on sex, age (+/- 2 years), and region. We applied the same exclusion criteria to controls as for cases.

Exposure

The exposure was defined as plasma donation rate. As the most appropriate exposure window is unknown, we defined several exposure windows:

1) A donation within 2 years before index date: no (reference) vs. yes.
2) A donation within 6 months before index date: no (reference) vs. yes.
3) A donation rate within 6 months before index date: 0 (reference group) vs. 1, 2, ≥3 donations.
4) History of a donation within 2 years before index date (to ensure that the donor was an active donor):

Category 0: no donation within 2 years (reference group),

Category 1: at least 1 donation within 2 years, but no donation within 6 months,

Category 2: at least 1 donation within 2 years and 1 donation within 6 months,

Category 3: at least 1 donation within 2 years and ≥ 2 donations within 6 months.

Furthermore, we explored more narrower exposure windows:

5) A donation more than 3 months and less than 2 years before index date:
    - No (reference) vs. yes,
    - 0 (reference) vs. 1, 2, ≥ 3 donations.
6) A donation within 1 month before index date:
    - No (reference) vs. yes,
    - 0 (reference) vs. 1, ≥ 2 donations.
7) A donation within 2–3 months before index date:
    - No (reference) vs. yes,
    - 0 (reference) vs. 1, 2, ≥ 3 donations.

Statistical analyses

Conditional logistic regression models were used to estimate odds ratios as a measure of the incidence rate ratios (ORs) and 95% likelihood ratio-based confidence intervals (CIs).

Two models were fitted: an unadjusted model with only matching variables (sex, age, and region), and an adjusted model with further adjustments for the frequency of whole blood donations. The frequency of whole blood donations was defined according to the specific analysis. For example, in the analysis of plasma donation rate within 2 years before the case-control date, the frequency of whole blood donations was calculated for the same period.

Results

In total, 67,698 plasma donors were included, of whom 33,849 became cases. Characteristics of the cases and controls are shown in **Table S4**. Cases were almost equally distributed between females and males. More cases were observed in the younger age groups.

Table S4. Characteristics of cases and controls

	Cases	Controls[a]
**All**	33,849	33,849
**Sex**		
Female	16,597	16,597
Male	17,252	17,252
**Age at case-control date**		
17–35	16,221	16,221
36–50	10,175	10,175
51–60	5,588	5,588
61–67	1,865	1,865
**Region**		
Capital	6,082	6,082
Central Denmark	9,774	9,774
North Denmark	3,811	3,811
South Denmark	9,502	9,502
Zealand	4,680	4,680
**Whole blood donation frequency[b]**		
0	16,193	16,193
1	5,855	5,855
2	4,169	4,169
≥ 3	7,631	7,631
**Infection outcomes during follow-up**		
All-cause infection, defined by hospital diagnoses and prescriptions	33,849	-
Respiratory tract infections, defined by prescriptions	14,144	-
All-cause infections, defined by hospital diagnoses	5,100	-
All-cause infections, defined by prescriptions	33,310	-

Characteristics of the cases and controls. [a]Selected controls could become cases on a later date. [b]Within 2 years before case-control date. With matched controls and complete data.

Associations between plasma donation and all-cause infections are summarized in **Table S5**. A donation within 2 years and within 6 months was associated with an increased risk of all-cause infection. A dose-response trend was observed for donations within 6 months: donating once was associated with an elevated risk, while donating more frequently suggested a lower effects.

A history of a donation within 2 years increased the risk across all groups, though the estimates decreased with more donations, particularly for those with at least 1 donation within 2 years and ≥ 2 donations within 6 months (group 3).

Donations occurring more than 3 months but less than 2 years before the index date were not associated with infection. In contrast, donations within 1 month of the index date were associated with decreasing ORs, which was further lowered with more donations (≥ 2 donations).

Donations within 2–3 months showed no significant association with infection, and donation rates in this timeframe demonstrated no clear trends.

There was no marked difference between the crude and adjusted model.

Table S6 summarize the associations between plasma donation rate and respiratory tract infections. These findings were consistent with the trends observed in **Table S5**.

Associations between plasma donation rates and hospital diagnoses as well as antimicrobial prescriptions are summarized in **Table S7–8**. Although limited by insufficient statistical power, a trend toward lower ORs for infection with increasing donation rate was observed in several groups.

Conclusion

In this large, nested case-control study, we explored various aspects of plasma donation and its potential impact on infection. Our analysis revealed no consistent evidence linking plasma donation to infection. The findings appeared to be influenced by the internal healthy donor effect, with higher donation rates correlating with a lower estimate for infections in several analyses.

Table S5. Plasma donations rates and all-cause infections

Exposure type	Exposure vs. reference	Crude model[a] OR (95% CI)	Adjusted model[b] OR (95% CI)
Donation within 2 years	yes vs. no	1.19 (1.13–1.24)	1.17 (1.12–1.22)
Donation within 6 months	yes vs. no	1.04 (1.01–1.07)	1.04 (1.00–1.07)
Donation rate within 6 months	1 vs. 0<br>2 vs. 0<br>≥ 3 vs. 0	1.16 (1.11–1.21)<br>1.01 (0.96–1.06)<br>0.92 (0.88–0.96)	1.15 (1.11–1.20)<br>1.01 (0.96–1.06)<br>0.92 (0.88–0.96)
History of a donation within 2 years[c]	1 vs. 0<br>2 vs. 0<br>3 vs. 0	1.21 (1.15–1.27)<br>1.31 (1.25–1.39)<br>1.09 (1.03–1.14)	1.19 (1.13–1.25)<br>1.29 (1.22–1.36)<br>1.07 (1.01–1.12)
Donation more than 3 months but less than 2 years	yes vs. no	0.95 (0.92–0.98)	0.94 (0.91–0.97)
Donation rate more than 3 months but less than 2 years	1 vs. 0<br>2 vs. 0<br>≥ 3 vs. 0	1.05 (1.00–1.09)<br>1.02 (0.97–1.07)<br>0.88 (0.84–0.91)	1.03 (0.99–1.08)<br>1.01 (0.96–1.06)<br>0.87 (0.84–0.91)
Donation within 1 month	yes vs. no	0.89 (0.86–0.92)	0.89 (0.85–0.92)
Donation rate within 1 month	1 vs. 0<br>≥ 2 vs. 0	0.92 (0.88–0.95)<br>0.72 (0.65–0.79)	0.91 (0.88–0.95)<br>0.71 (0.65–0.78)
Donation within 2–3 months	yes vs. no	0.99 (0.96–1.02)	0.99 (0.96–1.02)
Donation rate within 2–3 months	1 vs. 0<br>2 vs. 0<br>≥ 3 vs. 0	1.02 (0.98–1.06)<br>0.95 (0.90–1.01)<br>0.84 (0.77–0.93)	1.02 (0.98–1.06)<br>0.95 (0.90–1.01)<br>0.85 (0.77–0.93)

Conditional logistic regression models were used to estimate odds ratios (ORs) and 95% likelihood ratio-based confidence intervals (CIs). Exposure types were accessed before index date. All-cause infections were defined using both hospital diagnoses and prescriptions. [a]Crude model without any adjustments. [b]Adjusted model adjusted for frequency of whole blood donations. [c]History of a donation within 2 years before index date: no donation within 2 years (reference group – category 0); at least 1 donation within 2 years but no donation within 6 months (category 1); at least 1 donation within 2 years and 1 donation within 6 months (category 2); at least 1 donation within 2 years and ≥ 2 donations within 6 months (category 3).

Table S6. Plasma donation rates and respiratory tract infections

Exposure type	Exposure vs. reference	Crude model[a] OR (95% CI)	Adjusted model[b] OR (95% CI)
Donation within 2 years	yes vs. no	1.04 (0.97–1.12)	1.03 (0.96–1.10)
Donation within 6 months	yes vs. no	0.98 (0.93–1.03)	0.97 (0.93–1.02)
Donation rate within 6 months	1 vs. 0<br>2 vs. 0<br>≥ 3 vs. 0	1.06 (1.00–1.13)<br>1.00 (0.92–1.07)<br>0.88 (0.82–0.94)	1.05 (0.99–1.12)<br>0.99 (0.92–1.07)<br>0.88 (0.82–0.94)
History of a donation within 2 years[c]	1 vs. 0<br>2 vs. 0<br>3 vs. 0	1.08 (1.00–1.17)<br>1.11 (1.03–1.21)<br>0.97 (0.90–1.05)	1.07 (0.99–1.16)<br>1.10 (1.01–1.19)<br>0.96 (0.89–1.03)
Donation more than 3 months but less than 2 years	yes vs. no	0.94 (0.89–0.99)	0.93 (0.88–0.99)
Donation rate more than 3 months but less than 2 years	1 vs. 0<br>2 vs. 0<br>≥ 3 vs. 0	0.99 (0.92–1.07)<br>1.04 (0.96–1.13)<br>0.88 (0.83–0.94)	0.99 (0.92–1.06)<br>1.03 (0.95–1.12)<br>0.88 (0.83–0.94)
Donation within 1 month	yes vs. no	0.84 (0.79–0.89)	0.83 (0.79–0.88)
Donation rate within 1 month	1 vs. 0<br>≥ 2 vs. 0	0.86 (0.82–0.92)<br>0.66 (0.57–0.77)	0.86 (0.81–0.91)<br>0.66 (0.57–0.77)
Donation within 2–3 months	yes vs. no	0.98 (0.93–1.03)	0.98 (0.94–1.03)
Donation rate within 2–3 months	1 vs. 0<br>2 vs. 0<br>≥ 3 vs. 0	1.03 (0.98–1.09)<br>0.92 (0.85–1.01)<br>0.77 (0.67–0.89)	1.03 (0.98–1.10)<br>0.92 (0.85–1.01)<br>0.77 (0.67–0.89)

Conditional logistic regression models were used to estimate odds ratios (ORs) and 95% likelihood ratio-based confidence intervals (CIs). Exposure types were accessed before index date. Respiratory tract infections were defined using relevant prescriptions of antibiotics. [a]Crude model without any adjustments. [b]Adjusted model adjusted for frequency of whole blood donations. [c]History of a donation within 2 years before index date: no donation within 2 years (reference group – category 0); at least 1 donation within 2 years but no donation within 6 months (category 1); at least 1 donation within 2 years and 1 donation within 6 months (category 2); at least 1 donation within 2 years and ≥ 2 donations within 6 months (category 3).

Table S7. Plasma donation rates and hospital diagnoses for infections

Exposure type	Exposure vs. reference	Crude model[a] OR (95% CI)	Adjusted model[b] OR (95% CI)
Donation within 2 years	yes vs. no	0.89 (0.81-0.98)	0.91 (0.83–1.01)
Donation within 6 months	yes vs. no	0.85 (0.78-0.92)	0.86 (0.80–0.94)
Donation rate within 6 months	1 vs. 0<br>2 vs. 0<br>≥ 3 vs. 0	0.94 (0.84-1.05)<br>0.80 (0.70-0.91)<br>0.80 (0.71-0.89)	0.96 (0.86–1.07)<br>0.82 (0.71–0.94)<br>0.81 (0.72–0.90)
History of a donation within 2 years[c]	1 vs. 0<br>2 vs. 0<br>3 vs. 0	0.98 (0.88-1.09)<br>0.93 (0.82-1.05)<br>0.79 (0.71-0.88)	0.99 (0.89–1.11)<br>0.95 (0.84–1.08)<br>0.81 (0.73–0.91)
Donation more than 3 months but less than 2 years	yes vs. no	0.89 (0.81-0.97)	0.90 (0.82–0.98)
Donation rate more than 3 months but less than 2 years	1 vs. 0<br>2 vs. 0<br>≥ 3 vs. 0	0.93 (0.82-1.04)<br>0.98 (0.85-1.13)<br>0.85 (0.77-0.93)	0.95 (0.84–1.07)<br>1.00 (0.87–1.15)<br>0.85 (0.78–0.94)
Donation within 1 month	yes vs. no	0.67 (0.60-0.74)	0.66 (0.60–0.73)
Donation rate within 1 month	1 vs. 0<br>≥ 2 vs. 0	0.68 (0.61-0.76)<br>0.60 (0.45-0.78)	0.67 (0.60–0.75)<br>0.59 (0.45–0.77)
Donation within 2–3 months	yes vs. no	0.86 (0.79-0.94)	0.86 (0.79–0.94)
Donation rate within 2–3 months	1 vs. 0<br>2 vs. 0<br>≥ 3 vs. 0	0.89 (0.80-0.98)<br>0.85 (0.73-0.99)<br>0.70 (0.55-0.90)	0.89 (0.80–0.99)<br>0.84 (0.72–0.98)<br>0.70 (0.55–0.90)

Conditional logistic regression models were used to estimate odds ratios (ORs) and 95% likelihood ratio-based confidence intervals (CIs). Exposure types were accessed before index date. [a]Crude model without any adjustments. [b]Adjusted model adjusted for frequency of whole blood donations. [c]History of a donation within 2 years before index date: no donation within 2 years (reference group – category 0); at least 1 donation within 2 years but no donation within 6 months (category 1); at least 1 donation within 2 years and 1 donation within 6 months (category 2); at least 1 donation within 2 years and ≥ 2 donations within 6 months (category 3).

Table S8. Plasma donation rates and prescriptions of antimicrobials

Exposure type	Exposure vs. reference	Crude model[a]<br>OR (95% CI)	Adjusted model[b]<br>OR (95% CI)
Donation within 2 years	yes vs. no	1.18 (1.13–1.24)	1.17 (1.11–1.22)
Donation within 6 months	yes vs. no	1.07 (1.03–1.10)	1.06 (1.03–1.10)
Donation rate within 6 months	1 vs. 0<br>2 vs. 0<br>≥ 3 vs. 0	1.18 (1.14–1.23)<br>1.05 (1.00–1.10)<br>0.95 (0.91–0.99)	1.18 (1.13–1.23)<br>1.05 (1.00–1.10)<br>0.95 (0.91–0.99)
History of a donation within 2 years[c]	1 vs. 0<br>2 vs. 0<br>3 vs. 0	1.17 (1.12–1.24)<br>1.32 (1.25–1.39)<br>1.10 (1.05–1.16)	1.16 (1.10–1.22)<br>1.30 (1.23–1.37)<br>1.09 (1.03–1.14)
Donation more than 3 months but less than 2 years	yes vs. no	0.94 (0.91–0.97)	0.93 (0.90–0.96)
Donation rate more than 3 months but less than 2 years	1 vs. 0<br>2 vs. 0<br>≥ 3 vs. 0	1.01 (0.97–1.06)<br>1.03 (0.97–1.08)<br>0.88 (0.84–0.91)	1.00 (0.96–1.05)<br>1.02 (0.97–1.07)<br>0.87 (0.84–0.91)
Donation within 1 month	yes vs. no	0.89 (0.86–0.92)	0.89 (0.85–0.92)
Donation rate within 1 month	1 vs. 0<br>≥ 2 vs. 0	0.91 (0.88–0.95)<br>0.73 (0.66–0.80)	0.91 (0.88–0.95)<br>0.72 (0.66–0.80)<br>0.72 (0.66–0.80)
Donation within 2–3 months	yes vs. no	1.01 (0.98–1.05)	1.01 (0.98–1.05)
Donation rate within 2–3 months	1 vs. 0<br>2 vs. 0<br>≥ 3 vs. 0	1.04 (1.00–1.08)<br>0.98 (0.92–1.03)<br>0.90 (0.81–0.99)	1.04 (1.00–1.08)<br>0.98 (0.93–1.04)<br>0.90 (0.82–0.99)

Conditional logistic regression models were used to estimate odds ratios (ORs) and 95% likelihood ratio-based confidence intervals (CIs). Exposure types were accessed before index date. [a]Crude model without any adjustments. [b]Adjusted model adjusted for frequency of whole blood donations. [c]History of a donation within 2 years before index date: no donation within 2 years (reference group – category 0); at least 1 donation within 2 years but no donation within 6 months (category 1); at least 1 donation within 2 years and 1 donation within 6 months (category 2); at least 1 donation within 2 years and ≥ 2 donations within 6 months (category 3).

The cohort study with cumulative exposure rate measured over a one-year follow-up period

In this section, we describe the cohort study with time-varying exposure, cumulatively measured over a one-year follow-up period.

Method

Study population

As described as in the cohort study in the main paper.

Eligibility criteria for study entry included: being a first-time plasma donor between 1 January 2016, and 30 April 2024, 17–67 years of age, a minimum of six months' residency in Denmark before the first plasma donation, no Charlson Comorbidity Index score recorded within five years before the first plasma donation, and no redeemed prescription for any infection within three months before the first plasma donation.

Exposure and outcomes

Exposure was defined as a time-dependent variable and cumulative measured over a one-year follow-up after the first plasma donation and defined as plasma donations as a continuous variable and as a categorical variable: 1 plasma donation during follow-up (reference group), 2–5 donations, 6–11 donations, and ≥ 12 donations during follow-up.

Outcomes were defined as in the main analyses.

Statistical analyses

Study participants were followed from the date of first plasma donation until either the analysed outcome event, death, emigration, prescriptions and hospital contacts that potentially can lead to exclusion from blood donation, or end of follow-up limited by the registers, which was 30 April 2024. Follow-up was terminated one year after inclusion.

Cox proportional hazards regression models were used to estimate hazard ratios (HRs) and corresponding 95% confidence intervals (95% CIs).

Two confounder models were used: a basic model adjusted for age at baseline (17–35 (reference group), 36–50, 51–60, and 61–67 years of age), sex, and frequency of whole blood donations two years before the first plasma donation. A fully adjusted model with further adjustments for residential distance from the plasma donation site and region (Capital, Central Denmark, North Denmark, South Denmark, and Zealand Region).

Residential distance from the plasma donation site was defined as time-varying covariate using municipality codes and categorized as 0–30 km (category 0) and more than 30 km (category 1).

Results

Cohort characteristics are shown in **Table S9**. In total, 49,680 first-time plasma donors were included in the study with a median follow-up time of 1 year (Q1, 0.64; Q3, 1). In total, 9,641 became cases.

As expected, males were more prone to donate than females.

Associations between cumulative plasma donation rate and outcomes, including all-cause infection, respiratory tract infections based on prescriptions, all-cause infection based on hospital diagnoses, and all-cause infection based on prescriptions are detailed in **Tables S10–13**. When analysed as a continuous variable, higher donations rates were associated with lower effect estimates. This trend was consistent across all additional analyses of donation rates and various outcomes. However, the analysis using hospital-based all-caused infections was limited by statistical power. Female donors who donated 12 or more times had the lowest estimates. Adjustments did not affect the results.

Conclusion

In this large cohort study, we found no association between higher plasma donation rates and increased infection risk. In contrast, the association with infection generally decreased in magnitude with increasing donation rate, suggesting a significant bias from the internal healthy donor effect.

Table S9: Characteristics of the first-time plasma donors

	Numbers	Percentages
**Total**	49,680	100
**Sex**		
Female	23,264	46.8
Male	26,416	53.2
**Age at first-time plasma donation**		
17–35	26,606	53.6
36–50	13,976	28.1
51–60	7,017	14.1
61–67	2,081	4.19
**Region**		
Capital	7,263	14.6
Central Denmark	14,716	29.1
North Denmark	6,286	12.7
South Denmark	13,560	27.3
Zealand	7,855	15.8
**Distance from plasma donation site**		
0 km	34,428	69.3
1–20 km	6,623	13.3
≥ 20 km	8,629	17.4
**Calendar year (year of first plasma donation)**		
2016	5,624	11.3
2017	3,642	7.33
2018	3,597	7.24
2019	3,714	7.48
2020	4,278	8.61
2021	5,416	10.9
2022	6,301	12.7
2023	6,875	13.8
2024[a]	7,662	15.4
**Whole blood donation 2 years before index date**		
0 donation	16,021	32.2
1 donation	9,274	18.7
2 donations	7,331	14.8
≥ 3 donations	17,054	34.3
**Infection outcomes during follow-up**		
All-cause infection, defined by hospital diagnoses and prescriptions	9,641	
Respiratory tract infections, defined by prescriptions	3,924	
All-cause infections, defined by hospital diagnoses	824	
All-cause infections, defined by prescriptions	9,354	

Numbers with percentages. [a]The study period concluded 90 days before end of follow-up (30 April 2024).

Table S10: Plasma donation rates and all-cause infections, overall and by sex

	Model 1[a] HR (95% CI)	Model 2[b] HR (95% CI)
**Plasma donations (continuous variable)**		
Overall	0.97 (0.96–0.98)	0.97 (0.96–0.98)
Female	0.96 (0.94–0.97)	0.96 (0.94–0.97)
Male	0.98 (0.97–1.00)	0.98 (0.97–1.00)
**Plasma donation rate (reference group: 1 donation)**		
**Overall**		
2–5 donations	0.90 (0.86–0.94)	0.90 (0.86–0.94)
6–11 donations	0.80 (0.74–0.87)	0.80 (0.74–0.87)
≥ 12 donations	0.75 (0.60–0.93)	0.76 (0.61–0.95)
**Female**		
2–5 donations	0.87 (0.82–0.92)	0.88 (0.83–0.93)
6–11 donations	0.73 (0.65–0.81)	0.73 (0.65–0.82)
≥ 12 donations	0.67 (0.48–0.95)	0.69 (0.49–0.97)
**Male**		
2–5 donations	0.95 (0.89–1.02)	0.95 (0.89–1.02)
6–11 donations	0.91 (0.81–1.02)	0.91 (0.81–1.02)
≥ 12 donations	0.84 (0.63–1.12)	0.86 (0.64–1.14)

Cox proportional hazard analyses were used to calculate hazard ratios (HRs) with 95% confidence intervals (95% CIs). All-cause infections were defined using both hospital diagnoses and prescriptions. [a]Model 1: adjusted for sex, age, and whole blood donation frequency within two years before the first donation. [b]Model 2: model 1 with further adjustments for residential distance from the plasma donation site and region.

Table S11: Plasma donation rates and respiratory tract infections, overall and by sex

	Model 1[a] HR (95% CI)	Model 2[b] HR (95% CI)
**Plasma donations (continuous variable)**		
Overall	0.97 (0.95–0.98)	0.97 (0.95–0.98)
Female	0.96 (0.94–0.99)	0.96 (0.94–0.99)
Male	0.97 (0.95–0.99)	0.97 (0.95–0.99)
**Plasma donation rate (reference group: 1 donation)**		
**Overall**		
2–5 donations	0.90 (0.84–0.96)	0.90 (0.84–0.96)
6–11 donations	0.80 (0.71–0.91)	0.80 (0.70–0.90)
≥ 12 donations	0.70 (0.49–0.99)	0.72 (0.51–1.02)
**Female**		
2–5 donations	0.87 (0.80–0.96)	0.87 (0.80–0.96)
6–11 donations	0.76 (0.64–0.91)	0.76 (0.64–0.91)
≥ 12 donations	0.69 (0.40–1.19)	0.71 (0.41–1.22)
**Male**		
2–5 donations	0.94 (0.85–1.04)	0.93 (0.84–1.03)
6–11 donations	0.85 (0.71–1.01)	0.84 (0.71–1.00)
≥ 12 donations	0.73 (0.46–1.14)	0.75 (0.48–1.19)

Cox proportional hazard analyses were used to calculate hazard ratios (HRs) with 95% confidence intervals (95% CIs). Respiratory tract infections were based on prescriptions of antibiotics. [a]Model 1: adjusted for sex, age, and whole blood donation frequency within two years before the first donation. [b]Model 2: model 1 with further adjustments for residential distance from the plasma donation site and region.

Table S12: Plasma donation rates and hospital diagnoses for infections, overall and by sex

	Model 1[a] HR (95% CI)	Model 2[b] HR (95% CI)
**Plasma donations (continuous variable)**		
Overall	0.99 (0.95–1.02)	1.00 (0.96–1.03)
Female	1.00 (0.95–1.05)	1.02 (0.97–1.07)
Male	0.97 (0.93–1.02)	0.98 (0.93–1.03)
**Plasma donation rate (reference group: 1 donation)**		
**Overall**		
2–5 donations	0.90 (0.77–1.04)	0.93 (0.80–1.08)
6–11 donations	0.96 (0.73–1.25)	1.02 (0.78–1.33)
≥ 12 donations	0.70 (0.31–1.58)	0.83 (0.37–1.88)
**Female**		
2–5 donations	0.85 (0.69–1.05)	0.92 (0.74–1.13)
6–11 donations	1.06 (0.72–1.56)	1.20 (0.81–1.76)
≥ 12 donations	0.65 (0.16–2.64)	0.81 (0.20–3.30)
**Male**		
2–5 donations	0.94 (0.76–1.17)	0.96 (0.78–1.19)
6–11 donations	0.87 (0.60–1.26)	0.90 (0.62–1.30)
≥ 12 donations	0.71 (0.26–1.95)	0.81 (0.30–2.22)

Cox proportional hazard analyses were used to calculate hazard ratios (HRs) with 95% confidence intervals (95% CIs). [a]Model 1: adjusted for sex, age, and whole blood donation frequency within two years before the first donation. [b]Model 2: model 1 with further adjustments for residential distance from the plasma donation site and region.

Table S13: Plasma donation rates and prescriptions of antimicrobials, overall and by sex

	Model 1[a] HR (95% CI)	Model 2[b] HR (95% CI)
**Plasma donations (continuous variable)**		
Overall	0.97 (0.96–0.98)	0.97 (0.96–0.98)
Female	0.95 (0.94–0.97)	0.96 (0.94–0.97)
Male	0.98 (0.97–1.00)	0.98 (0.97–1.00)
**Plasma donation rate (reference group: 1 donation)**		
**Overall**		
2–5 donations	0.90 (0.87–0.94)	0.91 (0.87–0.95)
6–11 donations	0.80 (0.73–0.86)	0.79 (0.73–0.86)
≥ 12 donations	0.75 (0.60–0.93)	0.76 (0.61–0.95)
**Female**		
2–5 donations	0.88 (0.83–0.93)	0.88 (0.83–0.93)
6–11 donations	0.71 (0.64–0.80)	0.72 (0.64–0.80)
≥ 12 donations	0.69 (0.49–0.97)	0.70 (0.50–0.99)
**Male**		
2–5 donations	0.96 (0.89–1.03)	0.95 (0.89–1.02)
6–11 donations	0.91 (0.81–1.03)	0.91 (0.81–1.03)
≥ 12 donations	0.83 (0.62–1.12)	0.84 (0.63–1.14)

Cox proportional hazard analyses were used to calculate hazard ratios (HRs) with 95% confidence intervals (95% CIs). [a]Model 1: adjusted for sex, age, and whole blood donation frequency within two years before the first donation. [b]Model 2: model 1 with further adjustments for residential distance from the plasma donation site and region.

Delineation of infections

Severe infections were ascertained by using relevant diagnostic codes in the Danish National Patient Register (DNPR), which was established in 1977. The DNPR covers all information on hospital admissions since 1977 and hospital-based out-patient care (ambulatory specialty clinics) since 1995. Discharge diagnoses were recorded based on the International Classification of Diseases, Tenth Revision (ICD-10) from 1994 to the present.[7] Both primary and secondary diagnoses were used in this study.

Mild infections treated by the general practitioner were captured using data from the Danish National Prescription Register (NPR). The NPR was established in 1994 and retains key information on all prescribed drugs dispensed from all pharmacies in Denmark. This information includes the type of drug according to Anatomical Therapeutic Chemical (ATC) classification system and the date of prescription.[8]

Main outcome

All-cause infections defined by hospital diagnoses for infection (ICD-10 codes in the DNPR) and redeemed prescriptions of antimicrobials (ATC codes in the NPR).

ICD-10 codes:

A000, A001, A009, A01, A010, A011, A012, A013, A014, A02, A020, A021, A022, A028, A029, A03, A030, A031, A032, A033, A038, A039, A04, A040, A041, A042, A043, A044, A045, A046, A047, A048, A049, A05, A050, A051, A052, A053, A054, A058, A059, A06, A060, A061, A062, A063, A063A, A064, A065, A066, A067, A068, A068A, A069, A07, A070, A071, A072, A073, A073A, A078, A078A, A078B, A078C, A079, A08, DA080, A081, A082, A083, A083A, A084, A085, A09, A090, A099, A15, A150, A151, A152, A153, A154, A155, DA156, A157, A158, A158A, A158B, A158C, A159, A16, A160, A161, A162, A162A, A162B, A162C, A163, A164, A165, A167, A168, A168A, A168B, A168C, A169, A17, A170, A171, A178, A179, A18, A180, A181, A182, A183, A183F, A183G, A183H, A184, A184A, A184B, A184C, A184D, A184F, A184G, A184H, A184I, A184J, A185, A186, A187, A187A, A188, A19, A190, A191, A192, A198, A199, A20, A200, A201, A202, A203, A207, A208, A209, A21, A210, A211, A212, A213, A217, A218, A219, A22, A220, A221, A222, A227, A228, A229, A23, A230, A231, A232, A233, A238, A239, A24, A240, A241, A242, A243, A244, A25, A250, A250A, A251, A259, A26, A260, A267, A268, A269, A27, A270, A278, A279, A28, A280, A281, A282, A282B, A288, A289, A30, A300, A301, A302, A303, A304, A305, A308, A309, A31, A310, A310A, A310B, A310C, A311, A311A, A311B, A318, A319, A32, A320, A321, A327, A328, A328A, A329, A33, A339, A34, A349, A35, A359, A36, A360, A360A, A361, A362, A362A, A363, A368, A369, A37, A370, A371, A378, A379, A38, A389, A39, A390, A391, A392, A392A, A393, A394, A395, A398, A399, A40, A400, A401, A402, A403,

A408, A409, A41, A410, A411, A411A, A412, A413, A414, A415, A415A, A418, A419, A419B, A419C, A42, A420, A421, A422, A427, A428, A429, A43, A430, A431, A438, A439, A44, A440, A441, A448, A449, A46, A469, A48, A480, A481, A482, A483, A484, A484A, A488, A488A, A49, A490, A491, A492, A493, A498, A499, A499A, A50, A500, A501, A502, A503, A504, A505, A505A, A505B, A505C, A506, A507, A509, A51, A510, A511, A512, A513, A513A, A513F, A514, A514A, A515, A519, A52, A520, A521, A521D, A522, A523, A527, A527A, A527C, A528, A529, A53, A530, A539, A54, A540, A540A, A540B, A540D, A540E, A540F, A541, A541A, A541B, A542, A543, A543A, A544, A545, A546, A548, A548B, A548G, A549, A55, A559, A56, A560, A560A, A560C, A560D, A560E, A561, A562, A563, A564, A568, A57, A579, A58, A589, A59, A590, A590A, A590C, A598, A599, A60, A600, A601, A601A, A601B, A601C, A609, A63, A630, A630B, A630C, A630D, A638, A638A, A64, A649, A65, A659, A66, A660, A661, A662, A663, A664, A665, A666, A667, A668, A669, A67, A670, A671, A672, A673, A679, A68, A680, A681, A689, A69, A690, A690A, A690B, A690C, A691, A691A, A691B, A691C, A691E, A691F, A691G, A692, A692A, A692D, A692F, A698, A699, A70, A709, A71, A710, A711, A719, A74, A740, A748, A749, A75, A750, A751, A752, A753, A759, A77, A770, A771, A772, A773, A778, A779, A78, A789, A79, A790, A791, A798, A798A, A798B, A799, A80, A800, A801, A802, A803, A804, A809, A81, A810, A811, A812, A818, A818A, A818B, A819, A819A, A82, A820, A821, A829, A83, A830, A831, A832, A833, A834, A835, A836, A838, A839, A84, A840, A841, A848, A849, A85, A850, A851, A852, A858, A858A, A86, A869, A87, A870, A870A, A870B, A871, A872, A872A, A878, A879, A88, A880, A881, A888, A89, A899, A92, A920, A921, A922, A923, A924, A928, A928A, A929, A93, A930, A931, A932, A938, A94, A949, A95, A950, A951, A959, A96, A960, A961, A962, A968, A969, A97, A970, A971, A972, A979, A98, A980, A981, A982, A983, A984, A985, A985A, A985B, A985C, A988, A99, A999, B00, B000, B001, B001A, B001B, B001C, B002, B002A, B002B, B002C, B003, B004, B004A, B005, B007, B008, B009, B01, B010, B011, B011A, B012, B018, B019, B02, B020, B020A, B021, B022, B023, B027, B028, B029, B03, B039, B04, B049, B05, B050, B051, B052, B053, B054, B058, B059, B06, B060, B068, B069, B07, B079, B079A, B079C, B079D, B08, B080, B080A, B080B, B081, B082, B083, B084, B085, B088, B088A, B088B, B09, B099, B099A, B15, B150, B159, B159A, B16, B160, B161, B162, B169, B169A, B17, B170, B171, B172, B178, B179, B18, B180, B181, B182, B188, B189, B19, B190, B199, B20, B200, B200A, B201, B202, B203, B204, B205, B206, B207, B208, B209, B21, B210, B211, B212, B213, B217, B218, B219, B22, B220, B220A, B221, B222, B227, B23, B230, B231, B232, B238, B238A, B24, B249, B25, B250, B251, B252, B258, B259, B26, B260, B261, B262, B263, B268, B269, B27, B270, B271, B278, B279, B30, B300, B301, B301A, B301B, B302, B303, B303A, B303B, B303C, B308, B308A, B309, B33, B330, B331, B332, B333, B334, B338, B34, B340, B341, B341A, B341B, B342, B343, B344, B348, B349, B349A, B35, B350, B350A, B350B, B350C, B351, B352, B353, B354, B354A, B355, B356, B356B, B358, B358A, B358B, B359, B36, B360, B361, B362, B363, B368, B368A, B369, B37, B370, B370A, B371, B372, B372A, B372B, B373, B373A, B373B, B373C, B374,

B375, B376, B377, B378, B378A, B378B, B378C, B379, B38, B380, B381, B382, B383, B384, B387, B388, B389, B39, B390, B391, B392, B393, B394, B395, B399, B40, B400, B401, B402, B403, B407, B408, B409, B41, B410, B411, B418, B419, B42, B420, B421, B427, B428, B429, B43, B430, B431, B432, B438, B439, B44, B440, B441, B441A, B441B, B442, B447, B448, B448A, B448B, B449, B45, B450, B451, B451B, B452, B453, B457, B458, B459, B46, B460, B461, B462, B463, B463A, B464, B465, B468, B468A, B469, B469B, B47, B470, B471, B479, B48, B480, B481, B482, B483, B484, B487, B488, B49, B499, B499A, B50, B500, B500A, B508, B509, B51, B510, B518, B519, B52, B520, B528, B529, B53, B530, B531, B538, B538A, B54, B549, B55, B550, B551, B552, B559, B56, B560, B561, B569, B57, B570, B571, B572, B572A, B573, B574, B575, B579, B58, B580, B581, B582, B583, B588, B589, B59, B599, B60, B600, B600A, B601, B602, B608, B608A, B64, B649, B65, B650, B651, B652, B653, B658, B659, B66, B660, B661, B662, B663, B664, B665, B668, B669, B67, B670, B671, B672, B673, B674, B675, B676, B677, B678, B679, B68, B680, B681, B689, B69, B690, B691, B698, B699, B70, B700, B701, B71, B710, B711, B718, B719, B72, B729, B73, B739, B74, B740, B741, B742, B743, B744, B748, B748B, B749, B75, B759, B76, B760, B761, B768, B769, B769A, B77, B770, B778, B779, B78, B780, B781, B787, B789, B79, B799, B80, B809, B81, B810, B811, B812, B813, B814, B818, B82, B820, B829, B83, B830, B830A, B831, B832, B833, B834, B838, B839, B85, B850, B851, B852, B853, B854, B86, B869, B869A, B869B, B87, B870, B871, B872, B873, B874, B878, B879, B88, B880, B881, B882, B883, B888, B888A, B888B, B888C, B888D, B889, B89, B899, B899A, B90, B900, B901, B902, B908, B909, B91, B919, B92, B929, B94, B940, B941, B942, B948, B949, B95, B950, B951, B952, B953, B954, B955, B956, B956A, B957, B958, B96, B960, B961, B962, B963, B964, B965, B966, B967, B968, B97, B970, B971, B972, B973, B974, B975, B976, B977, B978, B98, B980, B981, B99, B999, D733, D738C, D738D, D762, E060A, E060B, E236A, E321, G00, G000, G001, G002, G003, G008, G008A, G008B, G009, G009A, G01, G019, G02, G020, G021, G028, G03, G030, G031, G032, G038, G039, G04, G040, G040A, G041, G042, G042A, G042B, G048, G048A, G048B, G048C, G049, G049A, G049B, G049C, G049D, G05, G050, G050P, G050R, G050S, G051, G051U, G051V, G051X, G052, G052K, G052L, G052M, G052N, G058, G06, G060, G060A, G060B, G060C, G060E, G060F, G060G, G060H, G060I, G060J, G060K, G060L, G061, G061A, G061C, G061E, G061F, G061G, G062, G062A, G062C, G07, G079, G079A, G079B, G079J, G079K, G531, G630, G734, G734C, G940, H000A, H00B, H00D, H050, H050A, H050B, H050C, H050D, H050E, H050F, H440, H440A, H440B, H60, H600, H600A, H601, H601B, H602, H603, H603A, H603B, H603C, H604, H604A, H605, H605B, H608, H608A, H609, H610, H610A, H62, H620, H621, H622, H623, H624, H65, H650, H650A, H651, H651C, H651D, H652, H653, H654, H654C, H659, H66, H660, H660A, H661, H662, H663, H664, H669, H67, H670, H671, H678, H68, H680, H681, H70, H700, H701, H702, H708, H709, H730, H730A, H731, H750, H830, H940, I00, I009, I009A, I009B, I01, I010, I011, I011A, I012, I018, I018A, I019, I02, I020, I020A, I029, I30, I300, I301, I301A, I301B, I301C, I301D, I301E, I308, I309, I320, I321, I33, I330, I339, I38, I389, I39, I390, I391, I392, I393, I394, I398, I400,

I410, I411, I412, I430, I520, I521, I521C, J00, J009, J01, J010, J011, J012, J013, J014, J018, J019, J02, J020, J020A, J028, J029, J029A, J029B, J029C, J03, J030, J038, J039, J039A, J039B, J039C, J039D, J039E, J039F, J04, J040, J040A, J040B, J040C, J040D, J041, J042, J05, J050, J051, J06, J060, J068, J069, J09, J091, J091A, J091B, J10, J100, J101, J101A, J101B, J101C, J108, J108A, J11, J110, J111, J111A, J111B, J111C, J118, J118B, J118D, J12, J120, J121, J122, J123, J128, J128A, J129, J13, J139, J139A, J139B, J14, J149, J149A, J149B, J15, J150, J151, J152, J153, J154, J155, J156, J156A, J157, J158, J159, J16, J160, J168, J17, J170, J171, J172, J173, J178, J18, J180, J181, J182, J188, J189, J20, J200, J200A, J201, J201A, J202, J202A, J203, J203A, J204, J204A, J205, J205A, J206, J206A, J207, J207A, J208, J208A, J209, J209A, J21, J210, J211, J218, J219, J22, J229, J31, J310, J311, J312, J32, J320, J321, J322, J323, J324, J328, J329, J340, J340A, J340B, J340D, J340E, J340F, J340G, J340H, J340I, J340J, J350, J350, J36, J369, J37, J370, J370A, J370B, J370C, J371, J382A, J383C, J383D, J387B, J387F, J387G, J390, J390A, J390B, J390C, J391, J391A, J391B, J391C, J398A, J85, J850, J851, J852, J853, J86, J860, J869, K112, K112A, K112B, K112C, K112D, K113, K113A, K113B, K113C, K113D, K12, K120, K120A, K120B, K120C, K120D, K121, K121A, K121B, K121C, K122, K122A, K122B, K130A, K140, K140A, K140B, K140C, K140D, K209A, K230, K35, K352, K353, K353A, K353B, K358, K358A, K358B, K358C, K36, K369, K37, K379, K57, K570, K570A, K570B, K570C, K571, K571A, K571B, K572, K572A, K572B, K572C, K573, K573A, K573B, K573C, K573D, K573E, K573F, K574, K574A, K575, K578, K579, K579A, K61, K610, K610A, K610B, K611A, K611B, K612, K613, K614, K628I, K628L, K630, K65, K650, K650A, K650B, K650C, K650D, K650E, K650F, K650G, K650H, K650I, K650J, K650K, K650L, K650M, K650N, K650O, K650P, K658, K658A, K658B, K658C, K658E, K658F, K658G, K658H, K658I, K659, K67, K670, K671, K672, K673, K678, K750, K750A, K750B, K750C, K750D, K770, K810A, K858A, K930, L00, L009, L009A, L009B, L009C, L01, L010, L010A, L010B, L010C, L011, L02, L020, L020A, L020B, L020C, L021, L021A, L021B, L021C, L022, L022A, L022B, L022C, L022D, L022E, L022F, L022G, L022H, L022I, L022J, L022K, L022L, L022M, L022N, L022O, L022P, L022Q, L022R, L022S, L022T, L023, L023A, L023B, L023C, L024, L024A, L024B, L024C, L024D, L024E, L024F, L024G, L024H, L024I, L024J, L024K, L024L, L024M, L028, L028A, L028B, L028C, L029, L029A, L029B, L029C, L03, L030, L030A, L030B, L030C, L030D, L030E, L030F, L030G, L030H, L030I, L030J, L030K, L031, L031A, L031B, L031C, L031D, L031E, L031F, L031G, L031H, L031I, L032, L033, L033A, L033B, L033C, L033D, L033E, L033F, L038, L038A, L038B, L039, L04, L040, L040A, L040B, L040C, L041, L042, L042A, L043, L048, L049, L05, L050, L059, L08, L080, L080A, L080B, L080C, L081, L088, L088C, L088D, L089, L303, L738H, L88, L889, L889C, M00, M000, M000A, M000B, M001, M001A, M001B, M002, M002A, M002B, M008, M009, M01, M010, M011, M012, M013, M014, M015, M016, M018, M03, M030, M031, M031A, M032, M032A, M032B, M036, M461, M462, M463, M463A, M464, M465, M465A, M490, M491, M492, M493, M600, M600A, M608A, M608A1, M609, M630, M631, M632, M650, M651, M680, M680G, M680H, M710, M711, M726, M730, M731, M86, M860, M861, M862, M863, M864, M865, M865A,

M866, M868, M868A, M869, M869A, M869B, M869C, M900, M901, M902, N080, N109A, N109B, N109C, N12, N129, N136, N136A, N136B, N136C, N136D, N136E, N151, N151A, N151B, N160, N288D, N288E, N288F, N290, N291, N30, N300, N301, N302, N303, N303A, N304, N308, N308A, N308B, N308C, N308D, N308E, N308F, N308G, N308H, N308J, N308K, N309, N330, N340, N340B, N340C, N341, N342, N342A, N342B, N342F, N342G, N370, N390, N390A, N390B, N41, N410, N411, N412, N412A, N413, N413A, N418, N419, N45, N450, N450A, N450B, N450C, N459, N459A, N459B, N459C, N459D, N459E, N481, N481A, N481B, N481C, N481D, N482, N482A, N482B, N482C, N482D, N482E, N482F, N482G, N482H, N482I, N49, N490, N491, N491A, N491B, N491C, N491D, N492, N492A, N492B, N492C, N492D, N498, N498A, N498B, N498C, N498M, N499, N511, N511K, N511L, N512, N61, N619, N619A, N619B, N619E, N70, N700, N700A, N700B, N700C, N700D, N700E, N700F, N700G, N701, N701A, N701B, N701C, N701D, N701E, N701F, N701G, N701H, N709, N709A, N709B, N709C, N71, N710, N710A, N710C, N710D, N710E, N710F, N711, N711A, N711B, N711D, N711E, N711F, N719, N72, N729, N729A, N729B, N729C, N729D, N729E, N729F, N73, N730, N730A, N730B, N730C, N730D, N730E, N731, N731A, N731B, N731C, N731D, N731E, N732, N732A, N732B, N732C, N732D, N732E, N733, N733A, N734, N734A, N735, N735A, N736, N736A, N736B, N738, N738A, N738B, N738C, N739, N74, N740, N741, N741A, N742, N743, N744, N748, N75, N750, N751, N758, N758A, N758B, N758C, N759, N76, N760, N760A, N760B, N760C, N760D, N760E, N761, N761A, N761B, N761C, N761D, N761E, N762, N762A, N763, N763A, N763B, N764, N764A, N764B, N764C, N764D, N764E, N765, N766, N766A, N768, N768A, N768B, N768C, N77, N770, N771, N771P, N771Q, N771R, and N778.

ATC codes:

ATC codes defining antimicrobial outcome included antibacterials (J01x), antivirals (J05x), and antifungals commonly prescribed for fungal skin infections (D01AC02/D01AC52/D01AE15/D01BA02).

Secondary outcomes

Respiratory tract infections:

Prescriptions of antibacterials most commonly used against bacterial respiratory tract infections: phenoxymethylpenicillin (J01CE02), clarithromycin (J01FA09), and roxithromycin (J01FA06).[9]

All-cause infection defined by hospital diagnoses only: See main outcome.

All-cause infection defined by prescriptions only: See main outcome.

Negative control outcomes

Emergency department diagnoses

We investigated typically injuries to the upper and lower extremities, i.e., ankle fractures, dislocation, or sprains. Injuries that potential could be related to plasma donation fails (i.e., nerve and blood vessels injuries) were not included.

ICD-10 codes:

S43, S430, S431, S432, S433, S434, S434A, S435, S435A, S436, S437, S53, S530, S531, S531A, S534, S63, S630, S630B, S630C, S630D, S630E, S630F, S630G, S630H, S630I, S631, S631A, S631B, S631C, S631D, S631E, S631F, S631G, S631H, S631I, S631J, S631K, S631L, S631M, S631N, S632, S635, S636, S636A, S636B, S636C, S636D, S636E, S636F, S636G, S636H, S636I, S636J, S636K, S637, S73, S730, S731, S83, S830, S831, S831A, S836, S836A, S836B, S836C, S836D, S93, S930, S930B, S930C, S931, S931A, S931B, S931C, S931D, S931E, S931F, S931G, S931H, S931I, S931J, S933, S933C, S934, S934A, S934B, S934C, S934D, S934E, S935, S935A, S935B, S935C, S935D, S935E, S935F, S935G, S935H, S935I, S935J, S936, S936A, S936B, S936C, S936D, T03, T030, T030A, T030B, T031, T032, T033, T034, T038, T039, T092, T112, T112A, T112B, T132, T132A, T132B, T143, T143A, T143B, T143D.

References

1. Kaspersen KA, Pedersen OB, Petersen MS, Hjalgrim H, Rostgaard K, Møller BK, et al. Obesity and risk of infection: results from the danish blood donor study. Epidemiology. 2015;26:580–9.

2. Kjerulff B, Kaspersen KA, Dinh KM, Boldsen J, Mikkelsen S, Erikstrup LT, et al. Smoking is associated with infection risk in healthy blood donors. Clin Microbiol Infect. 2022;:S1198-743X(22)00534-1.

3. Brodersen T, Rostgaard K, Lau CJ, Juel K, Erikstrup C, Nielsen KR, et al. The healthy donor effect and survey participation, becoming a donor and donor career. Transfusion. 2023;63:143–55.

4. Ware J, Kosinski M, Keller SD. A 12-Item Short-Form Health Survey: construction of scales and preliminary tests of reliability and validity. Med Care. 1996;34:220–33.

5. Charlson ME, Pompei P, Ales KL, MacKenzie CR. A new method of classifying prognostic comorbidity in longitudinal studies: development and validation. J Chron Dis. 1987;40:373–83.

6. Gasparini A. comorbidity: An R package for computing comorbidity scores. JOSS. 2018;3:648.

7. Schmidt M, Schmidt SAJ, Sandegaard JL, Ehrenstein V, Pedersen L, Sørensen HT. The Danish National Patient Registry: a review of content, data quality, and research potential. Clin Epidemiol. 2015;7:449–90.

8. Pottegård A, Schmidt SAJ, Wallach-Kildemoes H, Sørensen HT, Hallas J, Schmidt M. Data Resource Profile: The Danish National Prescription Registry. Int J Epidemiol. 2017;46:798–798f.

9. Dansk Selskab for Almen Medicin. Klinisk vejledning 2024: Akutte luftvejsinfektioner. Available from: https://www.dsam.dk/vejledninger/luftvejsinfektioner. Last accessed 26 Jan 2026.
